# Immunoinformatics Approach to Design Novel Subunit Vaccine against the Epstein-Barr Virus

**DOI:** 10.1128/spectrum.01151-22

**Published:** 2022-09-12

**Authors:** Abu Tayab Moin, Rajesh B. Patil, Tahani Tabassum, Yusha Araf, Md. Asad Ullah, Hafsa Jarin Snigdha, Tawfiq Alam, Safwan Araf Alvey, Bashudev Rudra, Sohana Akter Mina, Yasmin Akter, Jingbo Zhai, Chunfu Zheng

**Affiliations:** a The State Key Laboratory of Reproductive Regulation and Breeding of Grassland Livestock, School of Life Sciences, Inner Mongolia University, Hohhot, China; b Department of Genetic Engineering and Biotechnology, Faculty of Biological Sciences, University of Chittagong, Chattogram, Bangladesh; c Department of Pharmaceutical Chemistry, Sinhgad Technical Education Society's, Sinhgad College of Pharmacy, Vadgaon (Bk), Maharashtra, India; d Biotechnology Program, Department of Mathematics and Natural Sciences, School of Data and Sciences, BRAC University, Dhaka, Bangladesh; e Department of Biotechnology, Bangladesh Agricultural University, Mymensingh, Bangladesh; f Department of Biotechnology and Genetic Engineering, Faculty of Biological Sciences, Jahangirnagar University, Savar, Dhaka, Bangladesh; g Department of Microbiology, School of Environmental and Life Sciences, Independent University Bangladesh; h Department of Genetic Engineering and Biotechnology, School of Life Sciences, Shahjalal University of Science and Technology, Sylhet, Bangladesh; i Department of Physics, University of Dhaka, Dhaka, Bangladesh; j Medical College, Inner Mongolia Minzu University, Tongliao, China; k Key Laboratory of Zoonose Prevention and Control at Universities of Inner Mongolia Autonomous Region, Tongliao, China; l Department of Microbiology, Immunology and Infectious Diseases, University of Calgary, Calgary, Alberta, Canada; Oklahoma State University, College of Veterinary Medicine

**Keywords:** Epstein-Barr virus, immunoinformatics, subunit vaccine, molecular dynamics simulation, envelope glycoproteins

## Abstract

Epstein-Barr virus (EBV) is a lymphotropic virus responsible for numerous epithelial and lymphoid cell malignancies, including gastric carcinoma, Hodgkin’s lymphoma, nasopharyngeal carcinoma, and Burkitt lymphoma. Hundreds of thousands of people worldwide get infected with this virus, and in most cases, this viral infection leads to cancer. Although researchers are trying to develop potential vaccines and drug therapeutics, there is still no effective vaccine to combat this virus. In this study, the immunoinformatics approach was utilized to develop a potential multiepitope subunit vaccine against the two most common subtypes of EBV, targeting three of their virulent envelope glycoproteins. Eleven cytotoxic T lymphocyte (CTL) epitopes, 11 helper T lymphocyte (HTL) epitopes, and 10 B-cell lymphocyte (BCL) epitopes were predicted to be antigenic, nonallergenic, nontoxic, and fully conserved among the two subtypes, and nonhuman homologs were used for constructing the vaccine after much analysis. Later, further validation experiments, including molecular docking with different immune receptors (e.g., Toll-like receptors [TLRs]), molecular dynamics simulation analyses (including root means square deviation [RMSD], root mean square fluctuation [RMSF], radius of gyration [Rg], principal-component analysis [PCA], dynamic cross-correlation [DCC], definition of the secondary structure of proteins [DSSP], and Molecular Mechanics Poisson-Boltzmann Surface Area [MM-PBSA]), and immune simulation analyses generated promising results, ensuring the safe and stable response of the vaccine with specific immune receptors after potential administration within the human body. The vaccine’s high binding affinity with TLRs was revealed in the docking study, and a very stable interaction throughout the simulation proved the potential high efficacy of the proposed vaccine. Further, *in silico* cloning was also conducted to design an efficient mass production strategy for future bulk industrial vaccine production.

**IMPORTANCE** Epstein-Barr virus (EBV) vaccines have been developing for over 30 years, but polyphyletic and therapeutic vaccines have failed to get licensed. Our vaccine surpasses the limitations of many such vaccines and remains very promising, which is crucial because the infection rate is higher than most viral infections, affecting a whopping 90% of the adult population. One of the major identifications covers a holistic analysis of populations worldwide, giving us crucial information about its effectiveness for everyone’s unique immunological system. We targeted three glycoproteins that enhance the virulence of the virus to design an epitope-based polyvalent vaccine against two different strains of EBV, type 1 and 2. Our methodology in this study is nonconventional yet swift to show effective results while designing vaccines.

## INTRODUCTION

Epstein-Barr virus (EBV), a ubiquitous gammaherpesvirus also referred to as human herpesvirus 4 (HHV4), is one the most common causative agents of infectious mononucleosis (IM) among adults around the world (1). This lymphotropic virus has continuously raised considerable attention due to its oncogenic properties and association with several human malignancies, including posttransplant lymphoproliferative diseases (PTLDs), Burkitt’s lymphoma (BL), gastric carcinoma (GC), Hodgkin’s lymphoma (HL), and nasopharyngeal carcinoma (NPC) (2, 3). Primarily transmitted through the saliva of infected hosts, EBV establishes lifelong latency within memory B lymphocytes of healthy immunocompetent individuals, limiting its gene expression pattern to one or two essential viral proteins only, thereby escaping immune surveillance (4, 5). Viral persistence within B lymphocytes may expand in response to corresponding cognate antigen, subsequently differentiating into plasma cells, where viral replication and reactivation may occur (4, 6). The role of EBV replication in viral persistence has yet to be elucidated. However, many viral proteins have been detected in host oropharyngeal epithelial cells that may indicate viral replication (6–9), and viruses within the oral cavity can be transmitted to a new susceptible host.

EBV is a member of the gammaherpesviridae subfamily 1 and consists of a large linear double-stranded DNA (dsDNA) genome of ~172 kbp encoding more than 80 genes (10). Most of the proteins encoded by the EBV genome are crucial for nucleotide metabolism, viral replication, and the formation of viral structural elements, such as nucleocapsid, tegument proteins, and the envelope. The viral genome contains some latent genes that are not translated during the lytic phase and some genes that are expressed during latency, including the six EBV nuclear proteins (EBVs) and the three latent membrane proteins (LMPs) (11). The virus persists in multiple circular episomes inside the infected cell nucleus (12). However, some evidence of the virus persisting as integrated DNA within the host chromosome exists (13).

Several polymorphisms across the viral genome are attributable to the formation of different variants of EBV. The most prevalent two subtypes of the virus are type 1 and type 2, which are distinguished by sequence changes in EBV nuclear antigens 2 (EBNA-2) and 3 (EBNA-3) (11, 14). Some unique variations across the coding sequence, such as EBV nuclear antigen 1 (EBNA-1), BamHI Z fragment leftward open reading frame 1 (BZLF-1), and latent membrane protein 1 (LMP-1), can characterize strain-specific changes of the virus (15). Based on these variations within the genome, a heteroduplex tracking assay (HTA) that can specifically identify each variant has been developed to analyze a 264-bp region within the carboxy terminus for LMP-1 (16), the only protein with oncogenic properties within the host.

EBV establishes a persistent infection in around 90% of the global adult population. Viral infection is primarily asymptomatic but can progress in self-limiting infectious mononucleosis or other complications (1). Every year, about 84,000 cases of gastric carcinoma, 29,000 cases of Hodgkin’s lymphoma, 78,000 cases of nasopharyngeal carcinoma, 2,000 cases of lymphoma in transplant recipients, and 7,000 cases of Burkitt lymphoma are found to have an association with EBV. Moreover, EBV is associated with approximately 9% of all gastric carcinomas, 90% of gastric lymphoepithelioma, 7% of adenocarcinomas, and 6% of gastric adenocarcinomas. More than 10% of seronegative children who receive a solid organ transplant can develop EBV posttransplant lymphoproliferative disease. Immunocompromised hosts, such as AIDS patients, are at a higher risk of developing EBV-associated malignancies (17). Additionally, in a study conducted in the United States, researchers found that EBV was associated with all but 1 of 801 cases of multiple sclerosis, making it 99.88% of people affected by multiple sclerosis. Epstein-Barr virus has symptoms such as glandular fever and generally affects teenagers and young adults. It also causes symptoms such as swollen glands and sore throat, and people usually get it once in their life (18).

Unfortunately, to date, there are no effective vaccines for EBV. Although prophylactic vaccines that aim to prevent primary infection with this ubiquitous virus have been in development for more than 30 years, clinical trials of therapeutic vaccines have been conducted for more than 10 years, but neither a prophylactic nor a therapeutic vaccine could be licensed. Most approaches to developing a vaccine against EBV use recombinant viral glycoprotein 350 (gp350), an antigen expressed on the viral capsid that mediates attachment to CD21 receptors on the B-cell surface, facilitating viral entrance on cells (19, 20). This protein is the primary target of the immune system after natural infection, making it a suitable candidate for vaccine development (21). According to a phase 2 trial examining the immunogenicity and safety of the monomeric gp350 vaccine, there was a significant reduction in the incidence of infectious mononucleosis but not in the rate of EBV infection (22) and failed to show substantial effectiveness in human trials. Moreover, another randomized, single-blind, placebo-controlled study of EBV peptide vaccine conducted on HLA B*801 EBV-seronegative young adults reported a probable decline in infectious mononucleosis. No reduction was observed in asymptomatic EBV infection (23). Hence, the need for more efficient vaccine development for EBV immunization has become urgent worldwide to combat the disease burden.

**FIG 1:**
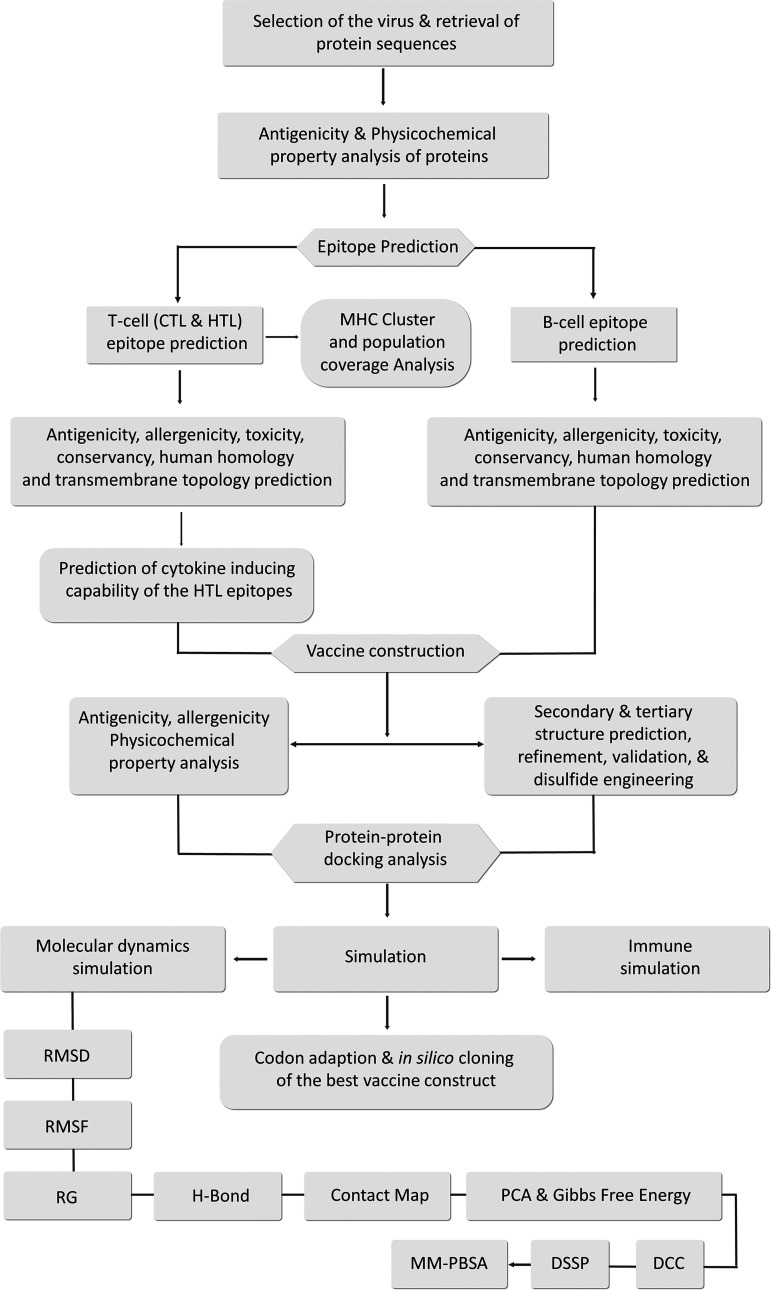
Flowchart depicting the step-by-step procedures used in the vaccine designing experiment with the list of the utilized tools.

This study has developed an effective multivalent vaccine following an immunoinformatics approach (Fig. 1) against two of the most virulent strains, strain B95-8 and strain AG876 from EBV type 1 and type 2, respectively. An epitope-based polyvalent vaccine was designed to produce substantial immune responses toward both EBV-1 and EBV-2, targeting the envelope glycoprotein B (gB), glycoprotein H (gH), and glycoprotein M (gM), respectively. Strain AG876 was used as the model during vaccine construction. gB, gH, and gM were targeted for T-cell and B-cell epitope prediction. The epitopes were screened over the criteria of full conservancy across both species and some other selection criteria. gB is crucial for viral fusion with epithelial cells and B cells, the cells within which the virus establishes lifelong persistence even in healthy immunocompetent hosts.

Along with gB, the fusion process requires the actions of gH for entry into epithelial cells (24). gH is also vital in regulating and triggering viral fusion with the epithelial cells. However, gM is a multispan, phosphorylated membrane protein that produces and releases progeny virions (25). Therefore, these three crucial proteins were chosen as potential targets to design the vaccine in this study. On administration, the vaccine is anticipated to inhibit these specific viral proteins and successfully attempt to prevent viral entry within target host cells, halting infection.

## RESULTS

### Strain identification and retrieval of the protein sequences.

Two unique EBV viral strains, EBV strain B95-8 and EBV strain AG876, were identified along with the three envelope glycoproteins for analysis from the NCBI database. The FASTA protein sequences of the three envelope glycoproteins, gB, gH, and gM, were retrieved from the UniProt database. Table 1 lists the UniProt accession numbers of the selected protein sequences.

**TABLE 1:** List of the proteins used in this study with their UniProt accession numbers

No.	Virus strain	Name of the protein	UniProt accession no.
1	EBV type 1 (strain B95-8)	Envelope glycoprotein B (gB)	P03188
2		Envelope glycoprotein H (gH)	P03231
3		Envelope glycoprotein M (gM)	P03215
4	EBV type 2 (strain AG876)	Envelope glycoprotein B (gB)	P0C763
5		Envelope glycoprotein H (gH)	Q1HVD2
6		Envelope glycoprotein M (gM)	Q1HVE9

### Antigenicity prediction and biophysical analyses of the proteins.

The pI, half-life, and grand average of hydropathicity (GRAVY) values were determined to assess the antigenicity of the vaccine construct and other physiochemical characteristics. These physiochemical property analyses resolved that the query glycoproteins gB, gH, and gM were all antigenic and had a half-life of 30 h in the mammalian cell culture system. The theoretical pI refers to the pH at which a protein remains uncharged. Aliphatic indexes were quite high in all the proteins. The aliphatic index of a protein is an indicator that deals with the relative volume of the amino acids in the side chain of the protein when the protein is occupied by the aliphatic amino acids, such as leucine, proline, and valine (26).

Envelope glycoprotein B showed the highest extinction coefficient (85,315 M^−1^ cm^−1^) and appeared as a hydrophilic protein with the lowest GRAVY value of −0.343. The GRAVY value determines if a compound is hydrophilic or hydrophobic. A negative GRAVY value indicates hydrophilic characteristics, suggesting that those molecules will be easily soluble in water. In contrast, a positive GRAVY value indicates the insolubility of the compound (27–29). All the results in the analysis were quite satisfactory. The results of the physicochemical property analysis are listed in Table S1 at https://drive.google.com/file/d/1xprLNGfmlJjYNR_X_MdCnhzfknwhpYvO/view?usp=drivesdk.

### Selecting the most promising epitopes.

T-cell and B-cell epitopes were predicted for vaccine construction, followed by physiochemical property assessment. Cytotoxic T cells can recognize antigens directly, whereas helper T cells assist in the activation of other immune cells, including B cells, macrophages, and cytotoxic T cells (30, 31). However, B cells can convert into antibody-producing plasma cells that act as humoral immune response mediators. The humoral immune response is not as robust as the cell-mediated immune response and weakens over time (32, 33), whereas the cell-mediated immune response provides stronger and lifetime immunity by secreting antiviral cytokines, specifically by identification and destruction of the infected cells (34, 35). The strain B95-8 of EBV type 1 was selected as the model for epitope determination. The topmost T-cell epitopes among the hundreds of epitopes generated by the server were screened for further analysis.

Similarly, B-cell epitopes that were longer than the length of 10 amino acids were selected for further screening. After selection, the T-cell and B-cell epitopes were screened, and the ones that displayed high antigenicity, were nonallergenic and nontoxic, were conserved across the selected strains, and showed nonhomology to the human proteome were finally selected for vaccine construction. Further, cytokine production ability of the helper T lymphocyte (HTL) epitopes was determined; the epitopes that might induce at least one cytokine were finally selected for vaccine construction. The conservancy across the selected strains particularly ensures the broad-spectrum activity of the vaccines over the selected EBV-1 and EBV-2. Furthermore, in the analysis of *N*-glycosylation sites of the selected proteins, none of the most promising epitopes were found at the potential glycosylation sites, as shown in Fig. S12 at https://drive.google.com/file/d/1xprLNGfmlJjYNR_X_MdCnhzfknwhpYvO/view?usp=drivesdk, implying that during posttranslational modification of the selected EBV glycoproteins, these antigenic epitopes are not supposed to be hidden by the glycans. Thus, the neutralizing antibodies (nAbs) should recognize them and take action so that viral infection and immunological escape can be prohibited.

Table 2 lists the most promising epitopes used in vaccine construction, antigenic, nontoxic, nonallergenic, conserved, and nonhomologous to the human proteome. Table S2 at https://drive.google.com/file/d/1xprLNGfmlJjYNR_X_MdCnhzfknwhpYvO/view?usp=drivesdk lists the potential T-cell epitopes of gB, Table S3 lists the potential T-cell epitopes of gH, and Table S4 lists the potential T-cell epitopes of gM with their respective topologies.

**TABLE 2:** List of the epitopes selected for vaccine construction (selection criteria: antigenicity, nonallergenicity, nontoxicity, conservancy, and nonhomology to the human proteome)

Protein name	MHC class I epitopes	MHC class II epitopes	B-cell epitopes
Envelope glycoprotein B (gB)	FLDKGTYTL	MSSIYGKAVAAKRLG	DIQCPSFGTRE
	ATVQIQFAY	SSIYGKAVAAKRLGD	DGKNKETFHERADSF
	TVMSSIYGK		NYKIVDYDNRGTNPQGE
	SQQPVQMLY		NTTVGIELPDAFKC
	FLTKKMTEV		DYHHFKTIELD
	VTDEGTSSF		SRDEQRASNVFDLE
	STIATETGK		
			
Envelope glycoprotein H (gH)	GLIGGATSV	VTFIISSDREVRGSA	PLEKQLFYYIGTMLPNTRPH
	MTAASYARY		SLEREDRDAWHLPAYKCVD
	GLYEERAHV		
			
Envelope glycoprotein M (gM)	TAMFPNLGY	ACGEVALIKARKKVS	DYGALNLTNYNLAHH
		VYITLVFIADCVAFI	FSPVWVVKAQDNSIPQDT
		YITLVFIADCVAFIY	
		CGEVALIKARKKVSG	
		YACGEVALIKARKKV	
		GEVALIKARKKVSGL	
		ITLVFIADCVAFIYY	
		YYACGEVALIKARKK	

### Population coverage analysis and cluster analysis of the major histocompatibility complex (MHC) alleles.

Results from the population coverage analysis demonstrated that the epitopes of the MHC class I alleles covered 90.77% of the world population, while the epitopes of the MHC class II alleles covered 86.40% of the world population. Combined, the epitopes of these two MHC classes covered 93.45% of the world population, on average, displaying a very high chance of effectiveness in administration across any part of the world. Among the countries noted here, Australia covered the highest percentage of the population responding to the MHC class I epitopes (83.57%), whereas North America covered the highest percentage of the population responding to the MHC class II epitopes (84.18%). Combined, Europe occupied the highest percentage of the population coverage for both the MHC class I and MHC class II epitopes (90.52%; Fig. S1 at https://drive.google.com/file/d/1xprLNGfmlJjYNR_X_MdCnhzfknwhpYvO/view?usp=drivesdk). From the population coverage analysis, it is clear that most of the world’s population possesses the target MHC alleles. Therefore, our designed vaccine should effectively combat the virus worldwide.

A cluster analysis of the MHC class I and MHC class II alleles that may interrelate with the three selected glycoproteins’ predicted epitopes was performed by using the online tool MHCcluster 2.0. Cluster analysis showed that all the alleles used in this study were phylogenetically related, as predicted by the MHCcluster 2.0 tool. Fig. S2 at https://drive.google.com/file/d/1xprLNGfmlJjYNR_X_MdCnhzfknwhpYvO/view?usp=drivesdk illustrates the results of the experiment, where the red zone represents a strong interaction, and the yellow zone indicates a weaker interaction.

### Multiepitope subunit vaccine construction.

**FIG 2:**
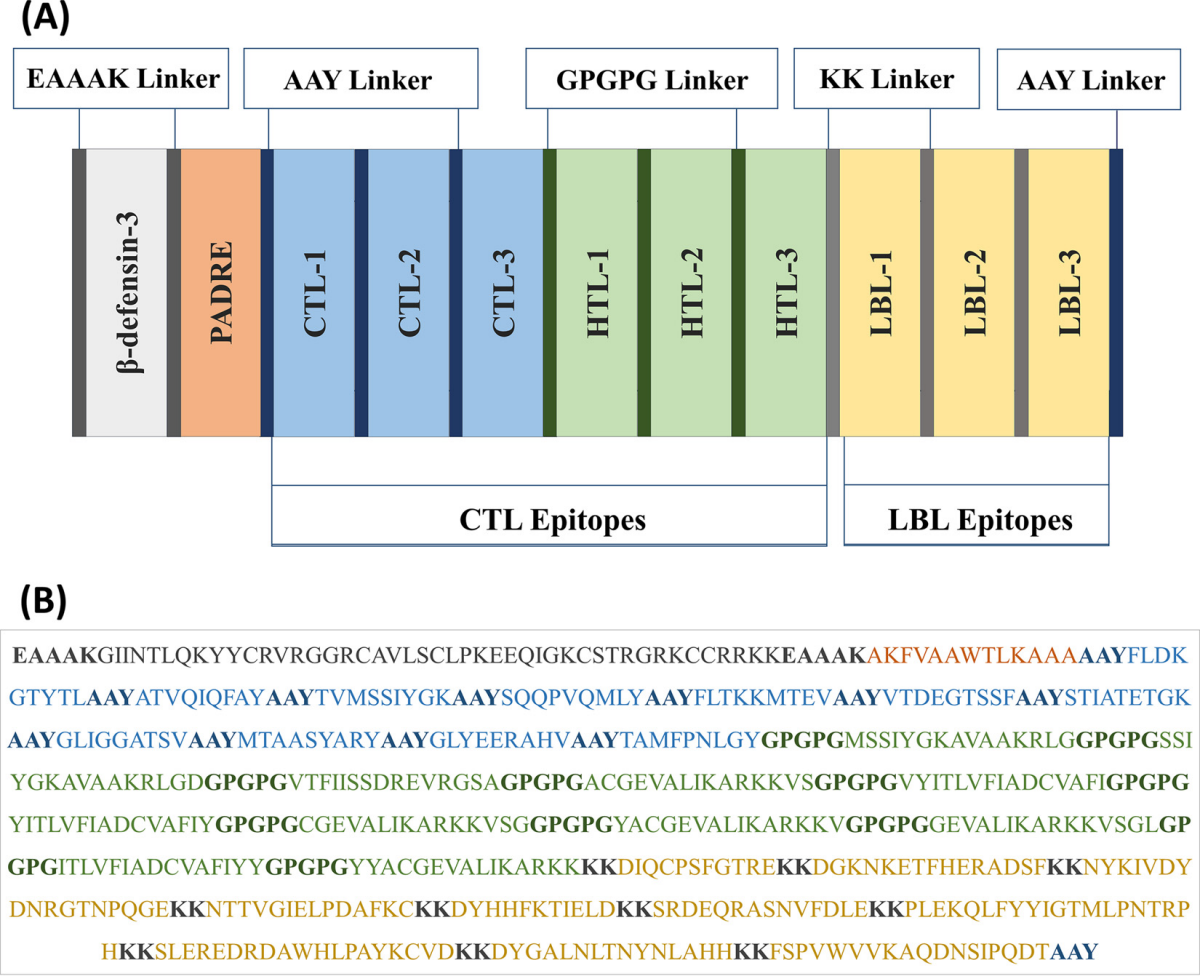
(A and B) Schematic representation (A) and sequence (B) of the possible vaccine constructs with linkers (EAAAK, AAY, GPGPG, and KK), adjuvant (human beta-defensin-3), PADRE sequence, and epitopes (CTL, HTL, and LBL) sequentially and appropriately.

As described in “Selecting the most promising epitopes,” the epitopes that were found to be highly antigenic, nonallergenic, nontoxic, conserved across the chosen strains of EBV, capable of inducing cytokines for HTL epitopes only in this case, and nonhomologous to the human proteome were considered the most promising epitopes and were subsequently chosen for vaccine construction. Most subunit vaccines require an adjuvant to induce immune reaction through the activation of innate immunity efficiently. Because antimicrobial peptides are great candidate molecules for stimulating innate and adaptive immune responses (36), the latest discovered human beta-defensins (hBds) were chosen as a suitable adjuvant for the vaccine. hBds are antimicrobial peptides that have an important role in innate immune responses at the epithelial barriers (37). Hence, they are used to conjugate the epitopes together to construct the vaccine. The PADRE sequence was associated with the adjuvant, which is reported to augment the activity of the vaccine. Along with the EAAAK linkers, AAY, GPGPG, and KK linkers were also used at their appropriate sites. The schematic presentation and the sequence of the final constructed vaccine are illustrated in Fig. 2.

### Antigenicity prediction and biophysical analyses of the vaccine construct.

The EBV vaccine was predicted to be highly antigenic and nonallergenic in the antigenicity and allergenicity test, suggesting that the vaccine might be able to produce the desired immune response in the human body without aggravating any undesirable allergenic reactions. Afterward, an analysis of other physiochemical properties of the EBV vaccine proved its competency. The vaccine appeared to be basic, possessing a pI value of 9.53. Additionally, the vaccine turned out to be stable due to an instability index (II) of 24.89, as an II of <40 is stable (35). The aliphatic index (AI) value, which measures the vaccine’s thermostability, was 73.18, indicating the vaccine’s stability within normal human body temperature. The vaccine proteins were hydrophilic due to their negative GRAVY value (−0.250). The half-life of the EBV vaccine was predicted at 30 h in mammalian reticulocytes and greater than 10 h in the Escherichia coli cell culture system, suggesting that mass production and purification of the vaccines should run smoothly in the E. coli cell culture system. The EBV vaccine also displayed high solubility after overexpression in E. coli cells. Solubility prediction using E. coli as a host is crucial to determine the postproduction processing of the vaccine. If the recombinant protein is not soluble, it may be nonfunctional due to inappropriate folding or the formation of many insoluble inclusions within the human body. It also represents easier vaccine purification during downstream postproduction processing (38). All these results point toward the suitability of the predicted vaccine as an effective preventive measure against EBV.

### Secondary structure prediction of the vaccine construct.

The secondary structure analysis conducted with PSI-blast based secondary structure PREDiction (PSIPRED), Garnier-Osguthorpe-Robson IV (GOR IV), Self-Optimized Prediction method With Alignment (SOPMA), and SIMPA96 servers presented promising results in amino acid percentages of α-helix, β-strand, and coil structure of the vaccine protein. The results showed distinction in the highest and lowest amino acid-containing structure and less deviation in the results of each of the α-helix, β-strand, and coil structures throughout the four servers used for analysis. For the EBV vaccine, the coil structure presented the highest percentages of amino acids (PRISPRED = 45.56%, GOR IV = 45.23%, SOPMA = 39.87%, and SIMPA96 = 50.67%). The second highest abundance of amino acids was found in the α-helix formation (PRISPRED = 36.52%, GOR IV = 39.70%, SOPMA = 31.99%, and SIMPA96 = 33.28%). Among the different predicted secondary structures, the β-strand contained the least amino acid percentages (PRISPRED = 16.92%, GOR IV = 15.08%, SOPMA = 21.61%, SIMPA96 = 15.89%; Fig. S3 and Table S5 at https://drive.google.com/file/d/1xprLNGfmlJjYNR_X_MdCnhzfknwhpYvO/view?usp=drivesdk).

### Protein three-dimensional (3D) structure prediction, refinement, and validation.

**FIG 3:**
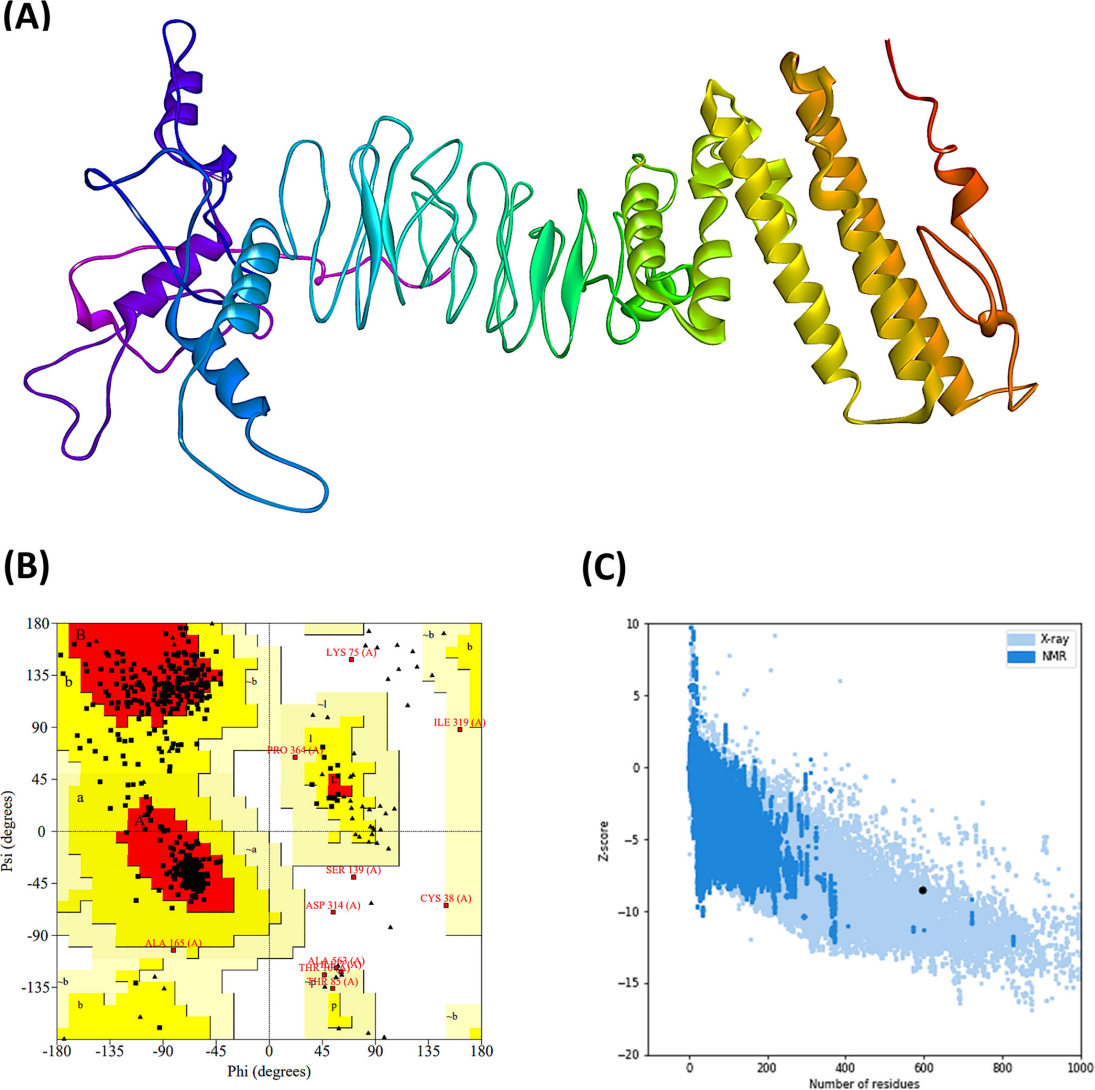
(A) Tertiary structure or 3D structure of the constructed vaccine EV. (B and C) Results of the Ramachandran plot analysis generated by the PROCHECK server (B) and quality score or Z-score graph generated by the ProSA-web server of the refined vaccine construct (C). In the Ramachandran plots, the orange and deep yellow-colored regions are the allowed regions, the light yellow regions are the generously allowed regions, the white regions are the outlier regions, and the glycine residues are represented as triangles.

The 3D structure of the vaccine protein was predicted by the RaptorX server and refined afterward to ensure that the protein would resemble the native or natural structure. Figure 3A represents the predicted 3D structure of the vaccine construct. After refining the protein structures generated by the RaptorX server, protein quality was validated by Ramachandran plots and Z-score, with satisfactory results obtained from both parameters. It was predicted from the Ramachandran plot analysis that amino acid residues of the EBV vaccine in the most favored regions were 83.2%, while in the additional allowed regions, the percentage was about 14.7%. However, the amino acid residues present in the generously allowed and disallowed regions were only about 1% of the total percentage (Fig. 3B), concluding that the quality of the predicted vaccine structure is quite good. Moreover, the Z-score of the EBV vaccine protein structure was −8.5, which is quite low. The low Z-score suggests a better quality of the query protein as it is bounded within the range of X-ray crystal structures that are experimentally proven and found in the Protein Data Bank (PDB; Fig. 3C).

### Vaccine protein disulfide engineering.

Protein disulfide engineering of the vaccine constructs predicted five pairs of amino acids that can form potential disulfide bonds with bond energy less than 2.2 kcal/mol. The five predicted amino acid pairs were Ser-27 and Ala-91, Ala-142 and Val-164, Ser-251, Ala-271, Ala-298, Val-316, Ser-501, and Glu-504. The selected amino acid pairs formed the mutant version of the original vaccines with disulfide bonds in the Disulfide by Design 2 v12.2 (DbD2) server (Fig. S4 at https://drive.google.com/file/d/1xprLNGfmlJjYNR_X_MdCnhzfknwhpYvO/view?usp=drivesdk). Because disulfide bonds confer stability to the proteins, the designed vaccine protein should be stable in the biological environment due to the potential to form five disulfide bonds among itself.

### Protein-protein molecular docking analysis.

Protein-protein docking is one of the most important steps in the vaccine designing process because the docking study reveals the interaction of vaccine protein with Toll-like receptors (TLRs). Because TLRs play important roles in triggering the proper immune response, the interaction of vaccines and TLRs is an important indication for proper protection against pathogens. Docking was performed by three different servers to increase the accuracy of the prediction. From the docking study, it is clear that the vaccine constructs generated sound and satisfactory results when docked with the TLRs by all the servers. The MM-GBSA analysis also showed good results, reflecting potential interaction between the vaccine construct and the TLRs (Table 3).

**TABLE 3:** Results of the molecular docking analysis using different servers

Target TLRs (with PDB IDs)	ClusPro energy score	Global energy (PatchDock server)	HawkDock score (the lowest score)	MM-GBSA (binding free energy, in kcal mol^−1^)
TLR-1 (6NIH)	−901.7	−22.06	−6,198.40	−83.81
TLR-2 (3A7C)	−1237.1	−24.33	−7,349.11	−114.90
TLR-3 (2A0Z)	−1,003.8	−33.31	−5,618.39	−91.45
TLR-4 (4G8A)	−897.6	−42.21	−5,901.67	−71.32
TLR-8 (3W3M)	−1,076.0	−2.20	−6,833.51	−101.17

### Conformational B-cell epitope prediction.

Following the docking study, the conformational B-cell epitopes of the EBV vaccine were predicted, which showed five potential regions with satisfactory scores ranging from 0.71 to 0.951, covering a total of 291 amino acids (Table S6 and Fig. S5 at https://drive.google.com/file/d/1xprLNGfmlJjYNR_X_MdCnhzfknwhpYvO/view?usp=drivesdk).

### Molecular dynamics (MD) simulation analyses.

The average, minimum, and maximum values of different MD simulation analyses (i.e., root mean square deviation [RMSD], root mean square fluctuation [RMSF], and radius of gyration [Rg] analyses) parameters are listed in Table 4.

**TABLE 4:** Average, minimum, and maximum values of different MD simulation analysis parameters

Value	TLR1	V-1^*a*^	TLR2	V-2^*a*^	TLR3	V-3^*a*^	TLR4	V-4^*a*^	TLR8	V-8^*a*^
RMSD (Å) in C-α atoms^*b*^										
Avg	2.53 (0.55)	15.52 (2.11)	4.83 (0.54)	19.72 (2.66)	2.86 (0.54)	20.91 (3.63)	2.05 (0.32)	24.13 (3.66)	2.31 (0.35)	22.19 (3.18)
Minimum	0.96	1.91	1.25	2.23	0.70	1.94	0.99	2.18	0.86	1.96
Maximum	4.54	19.28	6.39	23.21	4.36	29.26	3.59	28.51	3.13	26.49
RMSF (Å)^*b*^										
Avg	1.47 (0.53)	8.12 (3.08)	1.54 (0.63)	8.99 (3.71)	1.44 (0.54)	11.24 (5.30)	1.61 (0.60)	8.27 (3.90)	1.44 (0.79)	11.92 (4.60)
Minimum	0.68	3.99	0.69	3.83	0.62	2.70	0.66	2.84	0.59	3.72
Maximum	3.43	23.24	4.63	24.89	3.97	33.99	4.93	29.08	6.36	32.63
The radius of gyration (Å)^*b*^										
Avg	29.31 (0.32)	40.92 (2.23)	30.86 (0.31)	45.36 (2.63)	34.21 (0.262)	45.72 (2.29)	31.94 (0.39)	34.69 (2.08)	31.88 (0.12)	43.82 (2.20)
Minimum	28.35	36.61	29.64	38.00	33.56	37.99	30.61	32.08	31.41	37.66
Maximum	30.40	47.99	31.94	54.69	35.25	53.63	33.28	44.00	32.24	48.91

a V, vaccine chain.

b Standard deviations in average values are given in parentheses.

**FIG 4:**
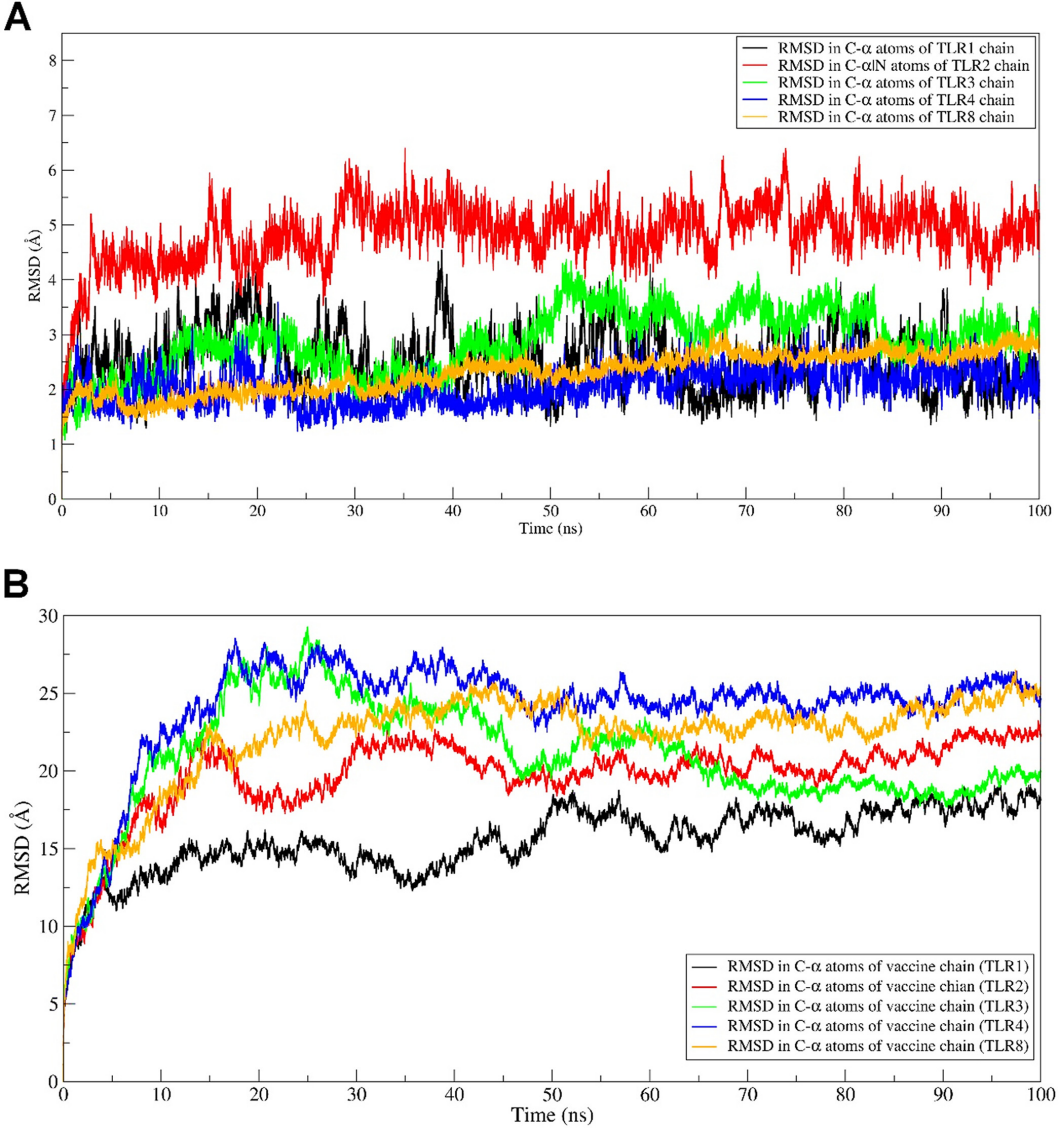

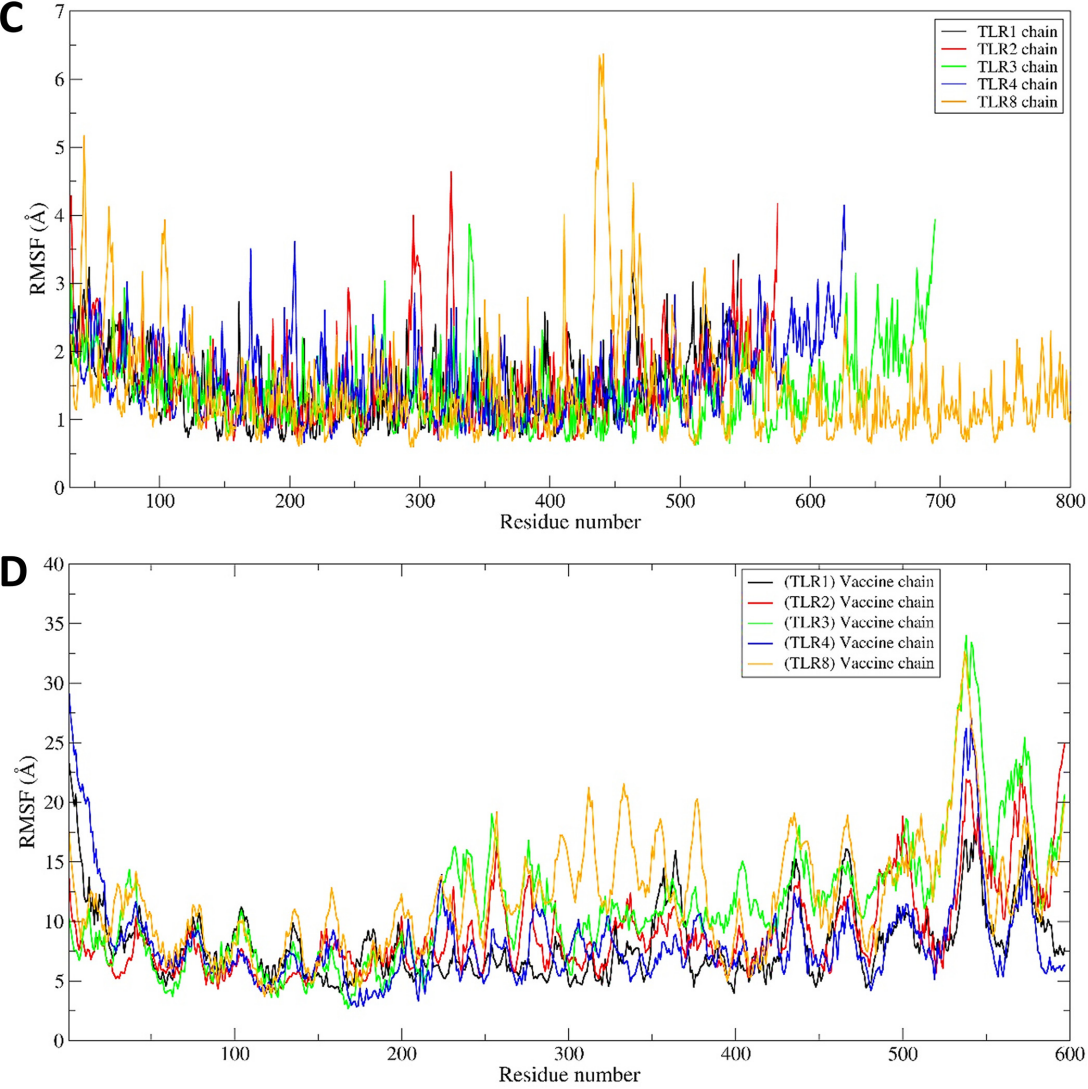

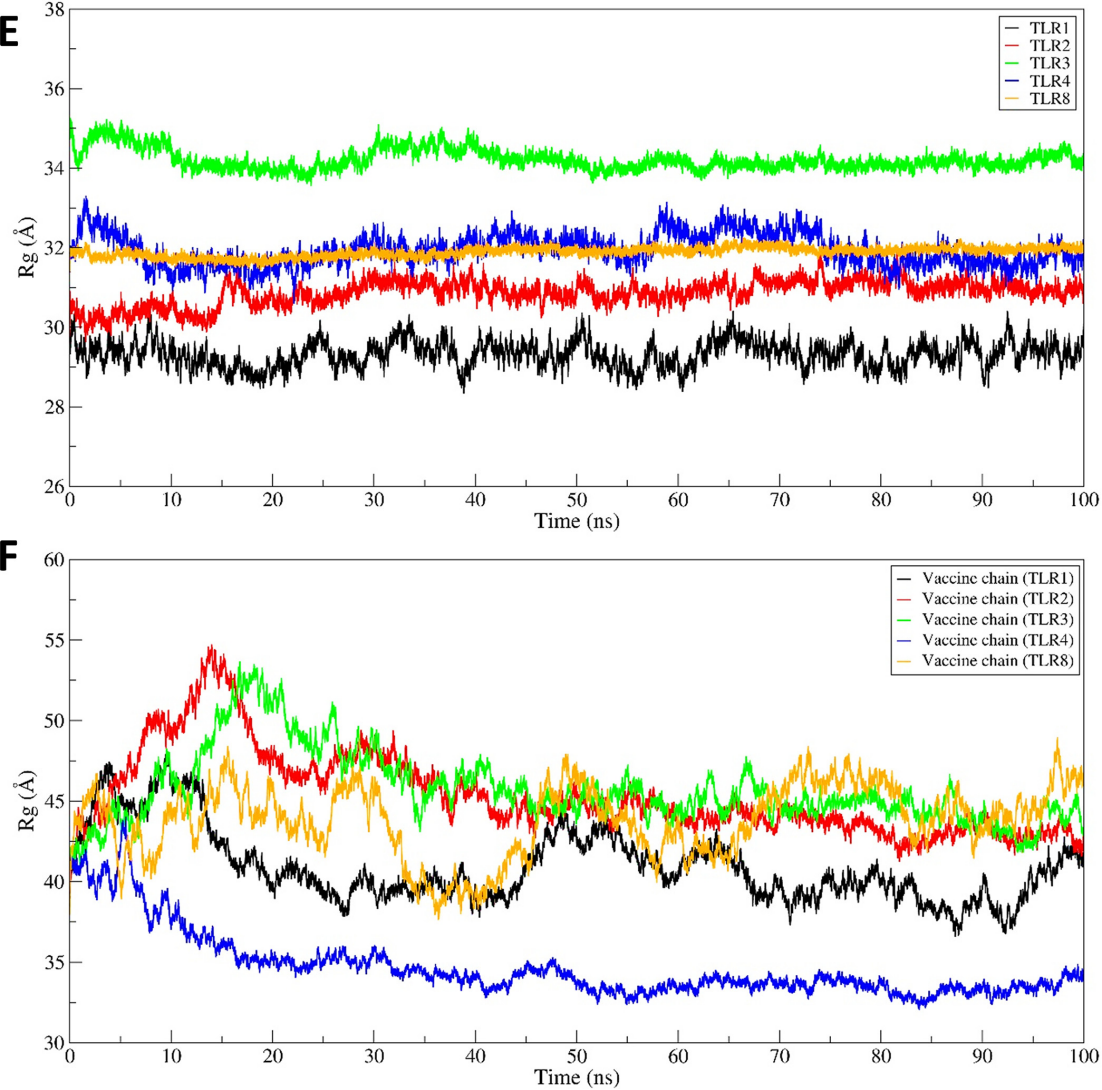
(A and B) RMSD against simulation time plots. The RMSD plot for the TLR chains (A) and the RMSD plot for the vaccine chain (B) are shown. (C and D) RMSF in residue atoms. RMSF for the TLR chain (C) and RMSF for the vaccine chain (D) are shown. (E and F) Total Rg plotted against simulation time. The Rg in the TLR chain (E) and Rg in the vaccine chain (F) are shown.

**(i) RMSD evaluation.** The RMSDs in C-α atoms of each TLR chain and vaccine chains (i.e., TLR1-vaccine complex, TLR2-vaccine complex, TLR3-vaccine complex, TLR4-vaccine complex, and TLR8-vaccine complex) were analyzed. The RMSD in vaccine chains has a higher magnitude of deviations than the RMSD in C-α atoms of TLR chains in respective systems (Fig. 4A and B). The RMSD in the TLR4 chain, with an average of 2.05 Å, was the largest among all TLRs. The lowest RMSD was observed with the TLR4 chain, and the RMSD for it was found quite stable compared to other TLR chains. However, major fluctuations were observed in TLR4 during the initial 25-ns MD simulation period, which stabilized until the end of the MD simulation. In the TLR1 chain, the initial fluctuations were observed until the 60-ns MD simulation period, which stabilized to an RMSD of around 2.53 Å. In the TLR2 chain, a larger magnitude of fluctuations than in other TLR chains was observed, with an average of 4.83 Å. For the TLR3 chain, the fluctuations in RMSD were gradually stabilized to an average of 2.86 Å. The RMSD in the TLR8 chain was found to be reasonably stable with the least fluctuations with an average of 2.31 Å.

The average RMSD in all TLRs was within 2.05 to 2.86 Å compared to the large magnitude of average RMSD in the vaccine chain, 15.52 to 24.13 Å. The RMSD in vaccines complexed with TLR1 remained almost stable during the initial 50-ns MD simulation with an average of around 15 Å. However, it slightly rises above an average of 15 Å for a brief period of 50 to 60 ns and stabilizes until the end of the simulation period. The RMSD in the vaccine chain complexed with TLR2 reached a peak of 23.21 Å at around 15 ns and, after that, remained stable with an average of 19.72 Å. The RMSD in the vaccine chain complexed with TLR3 showed the highest fluctuations among all the TLR vaccine chains. The peak RMSD of 29.26 Å reached around 25 ns and, after that, steadily lowered to an average of 20.91 Å. The highest magnitude of fluctuations was observed in the vaccine chain complexed with TLR4. In this case, the RMSD reached the maximum RMSD of 28.51 at around 15 ns and, after that, almost remained at an average of 24.31 Å. In the case of the vaccine chain complexed with TLR8, the RMSD steadily rose until 50 ns to a peak value of 26.49 Å and remained stable at an average of 22.19 Å.

**(ii) RMSF evaluation.** The RMSFs in the side chain atoms of each residue in corresponding TLRs and respective vaccine chains were evaluated. The results showed that the RMSF in all TLR chains was comparably lower than the RMSF in the vaccine chain for the corresponding TLR (Fig. 4C and D). The RMSFs in TLR1, TLR3, and TLR8 specifically had less magnitude of fluctuation with an average of 1.47, 1.44, and 1.44 Å, respectively. In TLR8, residues 400 to 500 showed significant fluctuations. However, the TLR1 chain residues showed the least magnitude of fluctuations compared to other TLRs. In the case of TLR2, residues in the range of 280 to 300 showed the largest magnitude of fluctuations.

Vaccine chains in complex with TLR8 showed major fluctuations in almost all residues, especially those 200 and beyond, which showed significant fluctuations. The RMSF in the vaccine chain complexed with TLR1 showed the lowest magnitude of fluctuations, with an average of 8.12 Å. The magnitude of RMSF was seemingly higher in the residues beyond 350 in the vaccine chain complexed with TLR1. A similar trend in RMSF in residues beyond 400 was seen in the vaccine chain complexed with TLR4, with an average of 8.27 Å. In the case of vaccine chain complexed with TLR2, a higher magnitude of fluctuations was observed for residues 200 to 300 and residues beyond 400, reaching peak fluctuation of 24.89 Å and an average of 8.99 Å. In the case of the vaccine chain complexed with TLR, the major fluctuations in residues were observed for residues beyond 200, with an average of 11.24 Å. The largest fluctuations were seen in residues beyond 200 in the vaccine chain complexed with the TLR8 chain, with a higher average value of 11.92 Å.

**(iii) Rg evaluation.** The Rg analysis provides insights into whether the protein under study remains folded and compact during the MD simulation. The overall results of the Rg analysis showed that the total Rg of TLR chains is comparably lower than the corresponding vaccine chains in each complex (Fig. 4E and F). Among all TLRs, the average total Rg for TLR1 was the lowest (29.31 Å). The TLR3 chain had the largest average Rg (34.21 Å) among all TLR chains. The Rg for the TLR2 chain was initially lower but rose at around 15 ns and after that remained stable, with an average of 30.86 Å. The average Rg for TLR4 and TLR8 was almost equivalent to 31.94 and 31.88 Å, respectively. However, the Rg in the TLR8 chain remained almost stable throughout the MD simulation compared to the slightly fluctuating Rg in the TLR4 chain.

The vaccine chain complexed with TLR4 had the lowest total Rg with an average of 34.69 Å, while the total Rg in vaccine chains in TLR2 and TLR3 had a higher total Rg with an average of 45.36 and 45.72 Å, respectively. In both the later vaccine chains complexed with TLR2 and TLR3, the Rg reached a peak at around 20 ns and, after that, steadily lowered until 40 ns and remained stable. For the vaccine chain complexed with TLR1, after an initial steep rise until 15 ns and again at around 50 ns, the Rg remained stable after 50 ns until the end of simulation, with an average of 40.92 Å. For the vaccine chain complexed with TLR8, the Rg deviated throughout the simulation period, with an average of 43.82 Å.

**(iv) Hydrogen-bond analysis.** The hydrogen bonds formed between the TLR and vaccine chains during the MD simulation were analyzed. Further, the initial equilibrated trajectory (0 ns) and trajectories extracted at 25, 50, 75, and 100 ns were investigated to find the residues from the TLR and vaccine chains involved in hydrogen-bond formation. The Hydrogen Bonds Computing Server (HBCS) (http://bioserver1.physics.iisc.ernet.in/hbcs/) was used to identify the residues involved in interchain hydrogen-bond formation. The results showed that the maximum number of hydrogen bonds was formed in the TLR3 complex with the vaccine chain (Fig. S6 at https://drive.google.com/file/d/1xprLNGfmlJjYNR_X_MdCnhzfknwhpYvO/view?usp=drivesdk). The TLR1-vaccine complex showed around 10 hydrogen bonds until the initial 30-ns MD simulation period and around 15 hydrogen bonds until the end of the simulation period.

While in the TLR2-vaccine complex, the initial 10-ns MD simulation showed around 5 hydrogen bonds and after that reached around 10 hydrogen bonds that formed consistently until the end of the simulation. In the TLR3-vaccine complex, around 10 hydrogen bonds formed during the initial 40-ns simulation period, steadily rising to 25 hydrogen bonds after around 50 ns. In this complex, after a 50-ns simulation period, the highest numbers of hydrogen bonds (around 35) were frequently formed. In the TLR4-vaccine complex, around 10 hydrogen bonds were formed during the initial 25-ns MD simulation period, which steadily rose to around 15 hydrogen bonds until 50 ns. For MD simulation period 50 to 80 ns, there was a steep lowering to 10 hydrogen bonds, followed by a steep rise to around 20 hydrogen bonds. In the last phase, after 80 ns, around 10 hydrogen bonds were formed. In the TLR8-vaccine complex, around 10 to 15 hydrogen bonds were consistently formed throughout the simulation.

**FIG 5:**
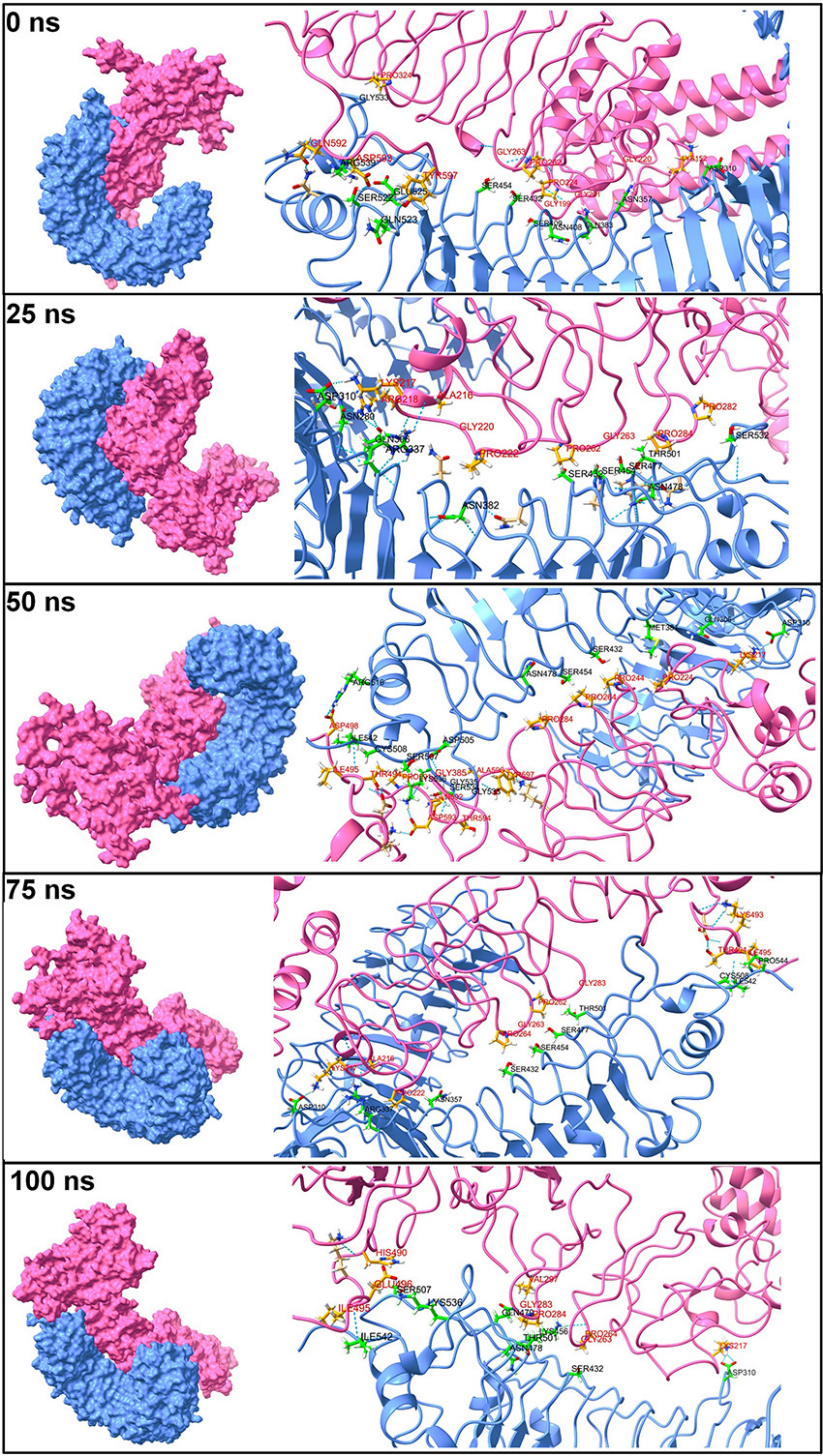
Interchain hydrogen bonds between TLR1 and vaccine. The surfaces on TLR1 and the vaccine chain are shown in light blue and light pink, respectively. The cartoon representations are shown in the same color for respective chains. The interacting residues of TLR1 are shown in the green stick representation, while the orange stick representation is for the vaccine chain. Labels are in black for the TLR1 chain and are in red for the vaccine chain. A similar color scheme is used for TLR chains and vaccine chains in Fig. 6 to 9.

The initial trajectory (0 ns) and trajectories isolated at 25, 50, 75, and 100 ns were subjected to the HBCS server to understand which residues participate in hydrogen-bond formation. In the TLR1-vaccine complex, the initial trajectory showed the hydrogen bonds between Gly-533, Arg-539, Ser-522, Glu-525, Gln-523, Ser-454, Ser-432, Ser-409, Asn-408, Gln-383, Asn-357, and Asp-310 residues from the TLR1 chain with Pro-324, Gln-592, Asp-593, Tyr-597, Gly-263, Pro-262, Pro-224, Gly-199, Gly-201, Gly-220, and Lys-152 residues of the vaccine chain (Fig. 5). At around 25 ns, the new hydrogen bonds between residues Asn-280, Gln-306, Arg-337, Asn-382, Ser-432, Ser-477, Thr-501, Asn-478, and Ser-532 from the TLR1 chain and residues Lys-217, Arg-218, Ala-216, Gly-220, Pro-222, Pro-284, and Pro-282 from the vaccine chain were formed in addition to the hydrogen bonds between Asp-310 and Ser-454 residues from the TLR1 chain and Pro-262 and Gly-263 residues from the vaccine chain. However, all these hydrogen bonds except a few breaks toward the 50-ns MD simulation period and new hydrogen bonds were formed between Arg-516, Ile-542, Cys-508, Ser-507, Asp-505, Lys-536, Gly-535, Ser-534, Gly-533, Asn-478, Ser-432, Met-381, Gln-306, and Asp-310 residues from the TLR1 chain and Asp-498, Ile-495, Thr-494, Pro-591, Gly-385, Gln-592, Asp-593, Thr-597, Pro-264, Pro-244, and Pro-224 residues from the vaccine chain. The hydrogen bonds between Asp-310, Ser-432, Ser-454, Cys-508, and Ile-542 residues from the TLR1 chain and Thr-494 and Ile-495 residues from the vaccine chain remained stable, while new hydrogen bonds with Arg-337, Asn-357, Ser-477, Thr-501, and Pro-544 residues from the TLR1 chain and Lys-217, Ala-216, Pro-222, Pro-264, Gly-263, Pro-262, Gly-283, and Lys-493 residues from the vaccine chain were formed during the 75-ns MD simulation period. Toward the end of MD simulations, most of these hydrogen bonds remained stable, such as the hydrogen bonds between Ile-542, Thr-501, Ser-432, and Asp-310 residues from the TLR1 chain and Ile-495, Gly-283, Gly-263, and Lys-217 residues from the vaccine chain, while most of the hydrogen bonds observed during the 50-ns simulation period were reformed.

**FIG 6:**
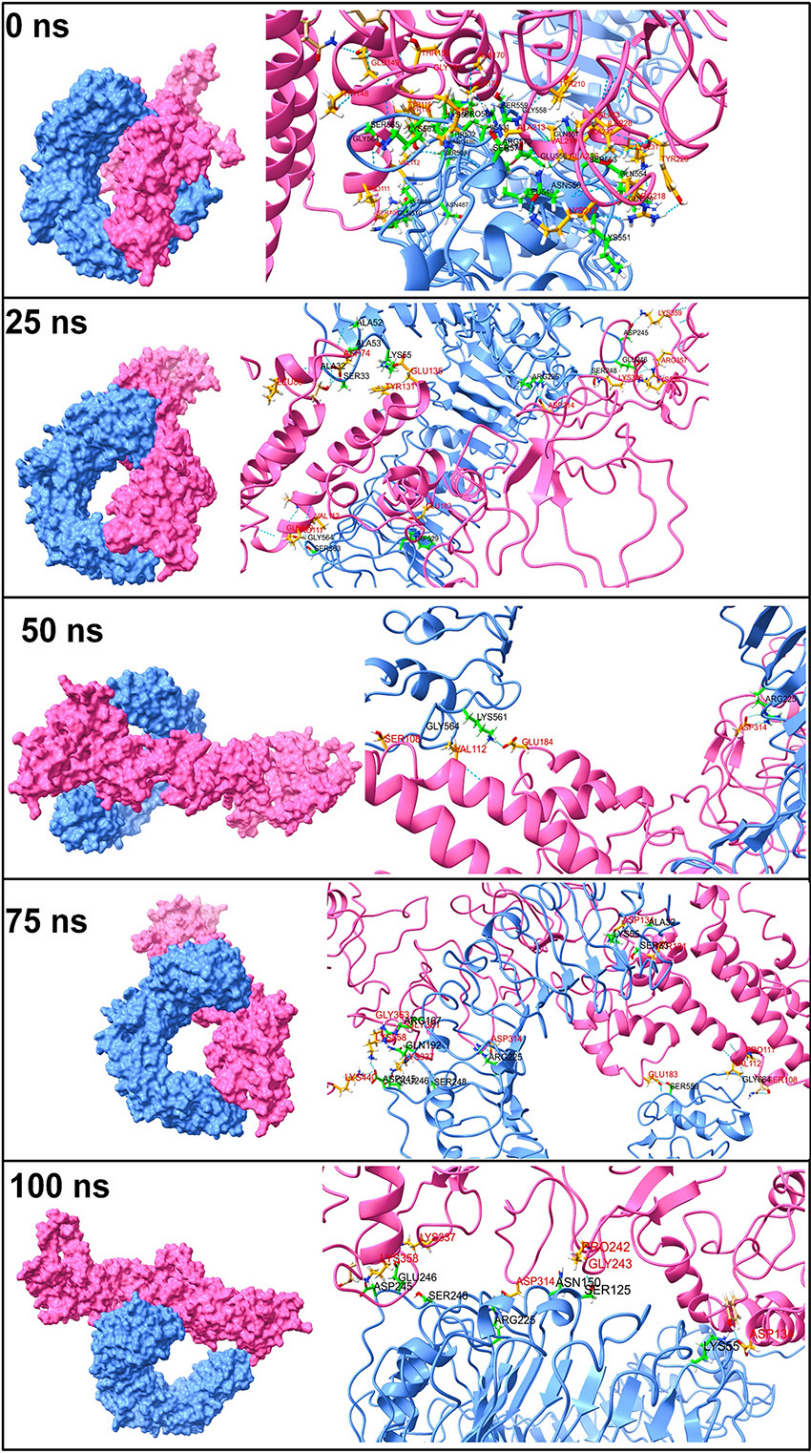
The interchain hydrogen bonds between TLR2 and vaccine.

The initial trajectory of the TLR2-vaccine complex showed many hydrogen bonds between various TLR2 chains and vaccine chains (Fig. 6). However, only a few of these hydrogen bonds remained intact during the next period of MD simulation. During 25 ns, the hydrogen bonds between Ala-52, Als-53, Ala-32, Ser-33, Lys-55, Gly-564, Ser-563, Trp-529, Arg-225, Ser-248, Glu-246, and Asp-245 residues from the TLR2 chain and Leu-80, Asp-74, Val-112, Gln-109, Pro-111, Tyr-131, Glu-135, Glu-183, Asp-314, Lys-326, Lys-359, Arg-357, and Lys-355 residues of the vaccine chain were formed. Most of these hydrogen bonds broke during the next 50-ns simulation period, and new hydrogen bonds were formed between the Lys-561 and Arg-225 residues of the TLR2 chain and Ser-108 and Glu-184 residues of the vaccine chain. Furthermore, during the 75-ns simulation period, new hydrogen bonds were formed between Arg-167, Gln-192, Asp-245, Glu-246, and Ser-248 from the TLR2 chain and the residues Gly-363, Gly-361, Lys-337, Lys-440, Glu-183, Asp-134, Tyr-131, and Val-112 from the vaccine chain. Most of these hydrogen bonds remained intact until the end of the simulation.

**FIG 7:**
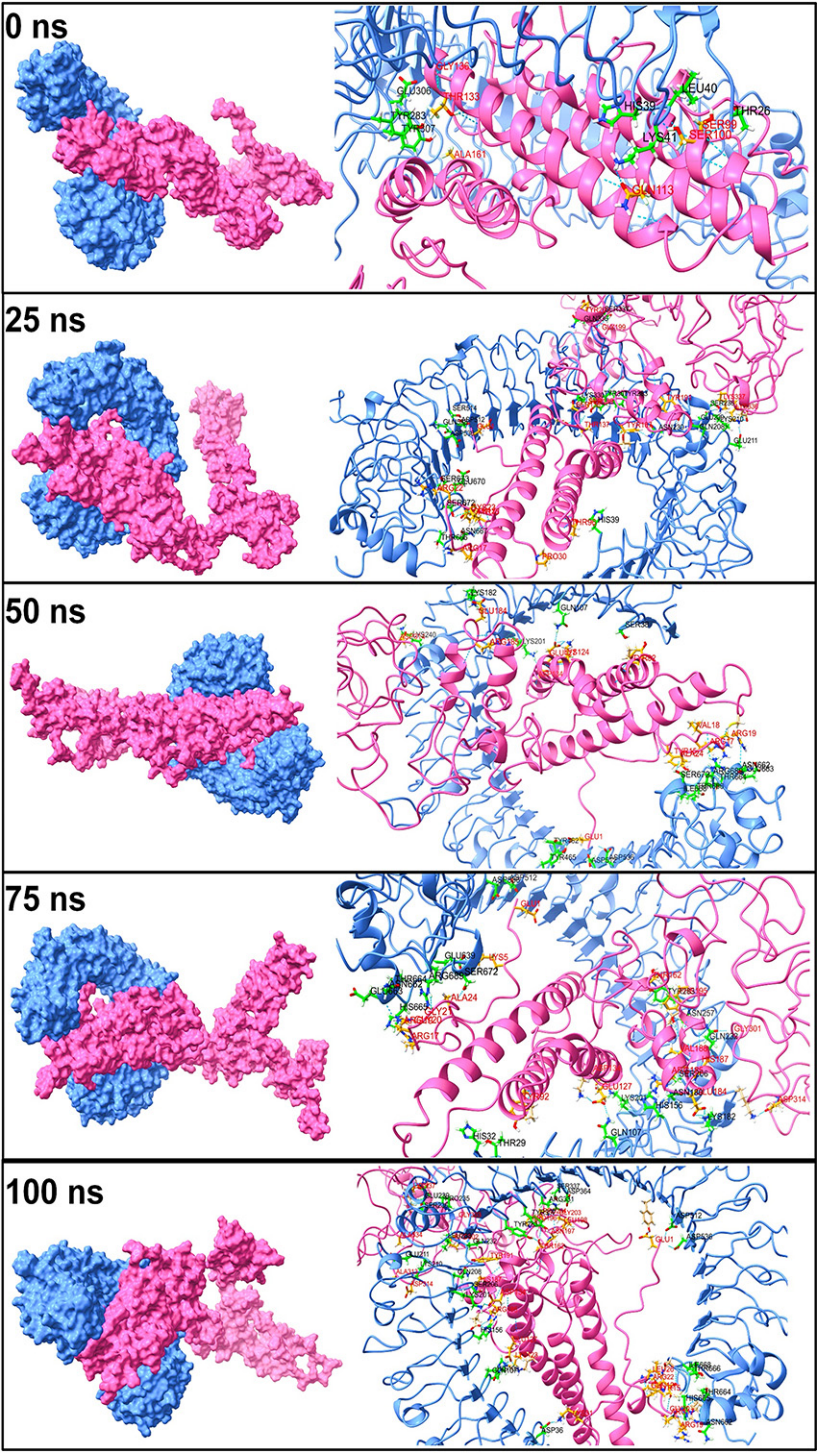
The interchain hydrogen bonds between TLR3 and vaccine.

The TLR3-vaccine complex showed the few hydrogen bonds between the Glu-306, Tyr-283, Tyr-307, His-39, Lys-41, Leu-40, and Thr-26 residues of the TLR3 chain and residues Gly-136, Thr-133, Ala-161, Gln-113, Ser-99, and Ser-100 of the vaccine chain in the initial trajectory (Fig. 7). However, all these hydrogen bonds broke during the 25-ns simulation period, and new hydrogen bonds were formed between Ser-337, Gln-336, Ser-514, Gln-538, Asp-512, Asp-536, Ser-673, Glu-670, Ser-672, Asn-667, Thr-666, Lys-330, Tyr-30, Tyr-283, Asn-230, Ser-236, Glu-239, Gln-208, and Glu-211 residues from the TLR3 chain and Glu-1, Arg-22, Cys-23, Tyr-26, Arg-17, Pro-30, Thr-36, Thr-137, Ser-136, Ser-139, Gly-199, Tyr-200, Tyr-167, Tyr-191, Lys-337, and Lys-336 residues of the vaccine chain. At around 50 ns, many new hydrogen bonds were formed, among which, the one between residues Asp-512, Asp-536, Thr-664, and Asn-662 of the TLR3 chain and residues Asp-314, Arg-185, Asp-134, Glu-1, Arg-17, and Arg-19 of the vaccine chain remained stable until the end of the simulation period.

**FIG 8:**
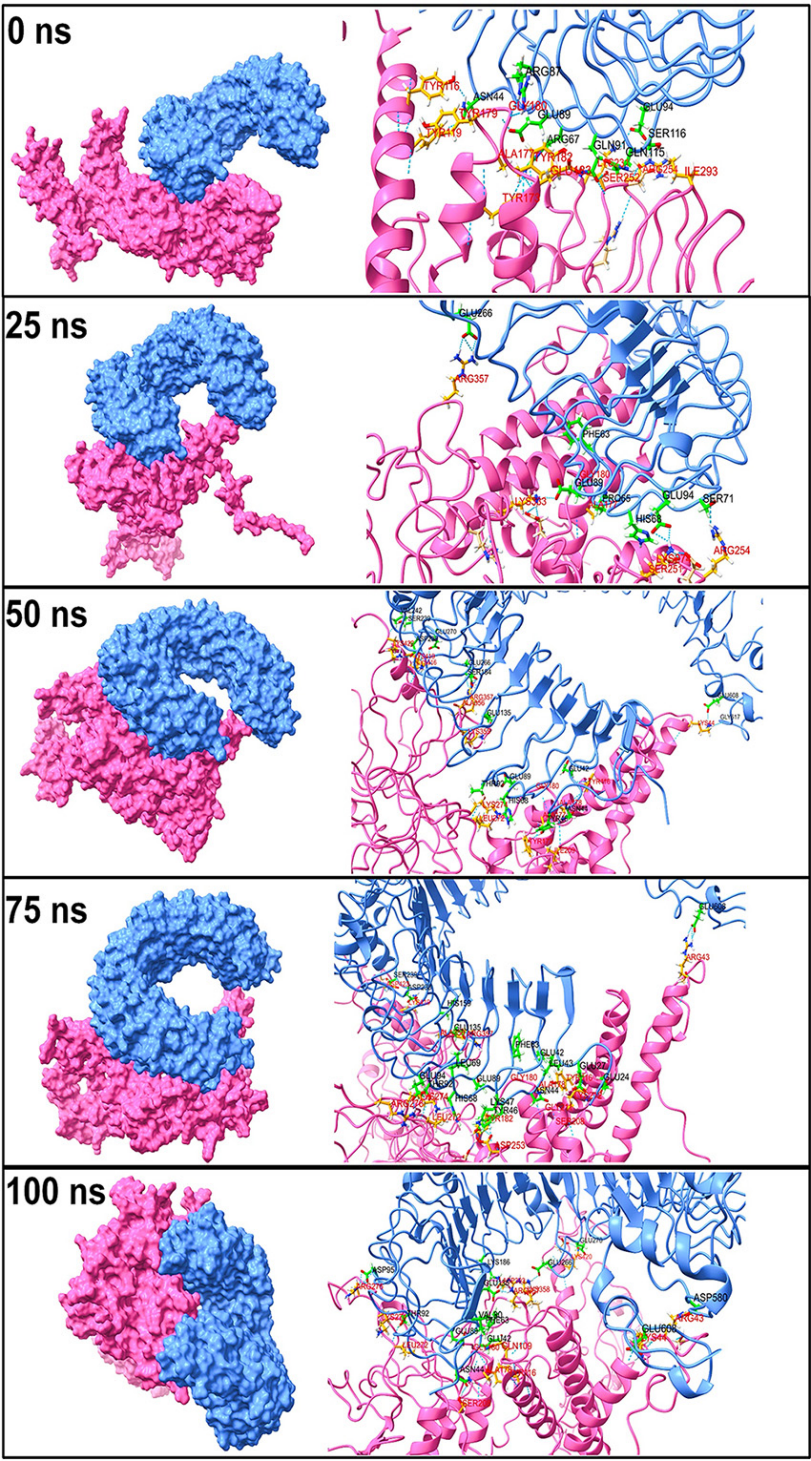
The interchain hydrogen bonds between TLR4 and vaccine.

The initial trajectory of the TLR4-vaccine complex showed the hydrogen bonds between residues Asn-44, Arg-87, Glu-89, Arg-67, Gln-91, Gln-115, Ser-116, and Glu-94 of the TLR4 chain and Tyr-116, Tyr-119, Tyr-179, Gly-180, Ala-177, Tyr-182, Glu-183, Tyr-173, Lys-231, Ser-252, Arg-254, and Ile-293 residues of the vaccine chain (Fig. 8). All of these hydrogen bonds except a hydrogen bond between Glu-89 from the TLR4 chain and Gly-180 from the vaccine chain broke during the 25-ns simulation period, and new hydrogen bonds between residues Glu-266, Phe-63, Pro-65, His-68, Glu-94, and Ser-71 of the TLR4 chain and Arg-357, Lys-333, Ala-177, Lys-274, Ser-251, and Arg-254 of the vaccine chain were formed. New interchain hydrogen bonds were formed during the simulation period after 25 ns until 75 ns. However, the hydrogen bonds between Ser-239, Asp-238, Glu-135, Thr-92, His-68, Glu-89, Tyr-46, Asn-44, Glu-42, and Glu-608 of the TLR4 chain and Ala-356, Arg-357, Lys-274, Leu-272, Gly-180, Ala-178, Tyr-116, and Ser-208 were seen formed consistently during this simulation period. During the 1ast 100-ns simulation period, as seen in the last 100-ns trajectory, few hydrogen bonds formed in the initial equilibrated trajectory were reformed, while few hydrogen bonds observed in the 75-ns trajectory remained stable.

**FIG 9:**
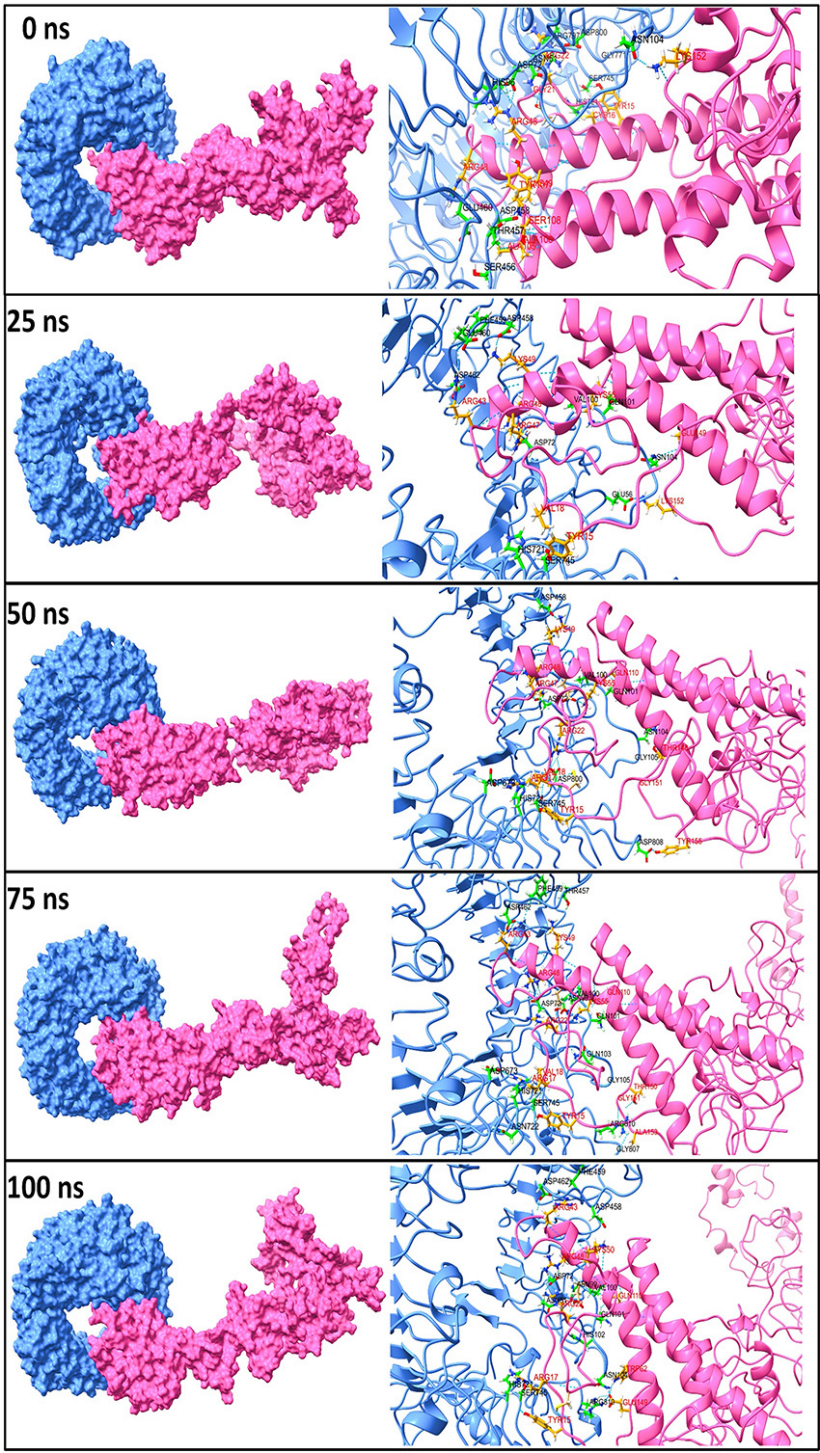
The interchain hydrogen bonds between TLR8 and vaccine.

The initial trajectory of the TLR8-vaccine complex showed the hydrogen bonds between Ser-745, Asn-104, Gly-771, Asp-800, Arg-797, Asn-51, Asp-72, His-96, Ser-745, His-721, Glu-460, Asp-458, Thr-457, and Ser-456 residues of the TLR8 chain and Tyr-15, Arg-22, Gly-21, Lys-152, Cys-16, Arg-48, Arg-43, Tyr-107, Ser-108, Ala-106, and Ala-105 residues of vaccine chain (Fig. 9). Among these, the hydrogen bonds with Ser-745, His-721, Glu-460, and Asp-458 residues of the TLR8 chain and Lys-152, Tyr-15, Arg-48, and Arg-43 remained stable until the 25-ns simulation period along with few other hydrogen bonds. At 50 ns, among the newly formed hydrogen bonds, the one with Val-100, Gln-101, Asp-72, Gln-101, and Asn-104 of the TLR8 chain and Lys-49, Arg-48, Lys-55, Arg-47, and Val-18 of the vaccine chain remained stable, and further, few more new hydrogen bonds were formed. Most hydrogen bonds remained stable, as seen in the trajectory extracted at 75 ns. Further, some hydrogen bonds formed between Phe-459, Asp-462, Asp-72, Ser-745, and Arg-810 of the TLR8 chain, and Arg-43, Arg-48, Arg-22, Arg-17, and Tyr-15 of the vaccine chain remained stable until the end of the simulation period.

**(v) Contact map analysis.** Contact map analysis was performed to assess the extent of residue-to-residue contacts in TLR-vaccine complexes. The results are shown Fig. S7 at https://drive.google.com/file/d/1xprLNGfmlJjYNR_X_MdCnhzfknwhpYvO/view?usp=drivesdk. The contact maps show that the key residue-to-residue contact in individual chains (i.e., TLR or vaccine chain) follows a unique pattern. The TLR chain shows a similar pattern of residue-to-residue minimum distances during the entire simulation. However, the vaccine chain showed a slightly varied pattern of minimum residue-to-residue distances. There were more interchain residue contacts in the TLR3-vaccine complex and fewer in the TLR2-vaccine complex. The residues involved in establishing the interchain contacts varied in each TLR-vaccine complex.

**FIG 10:**
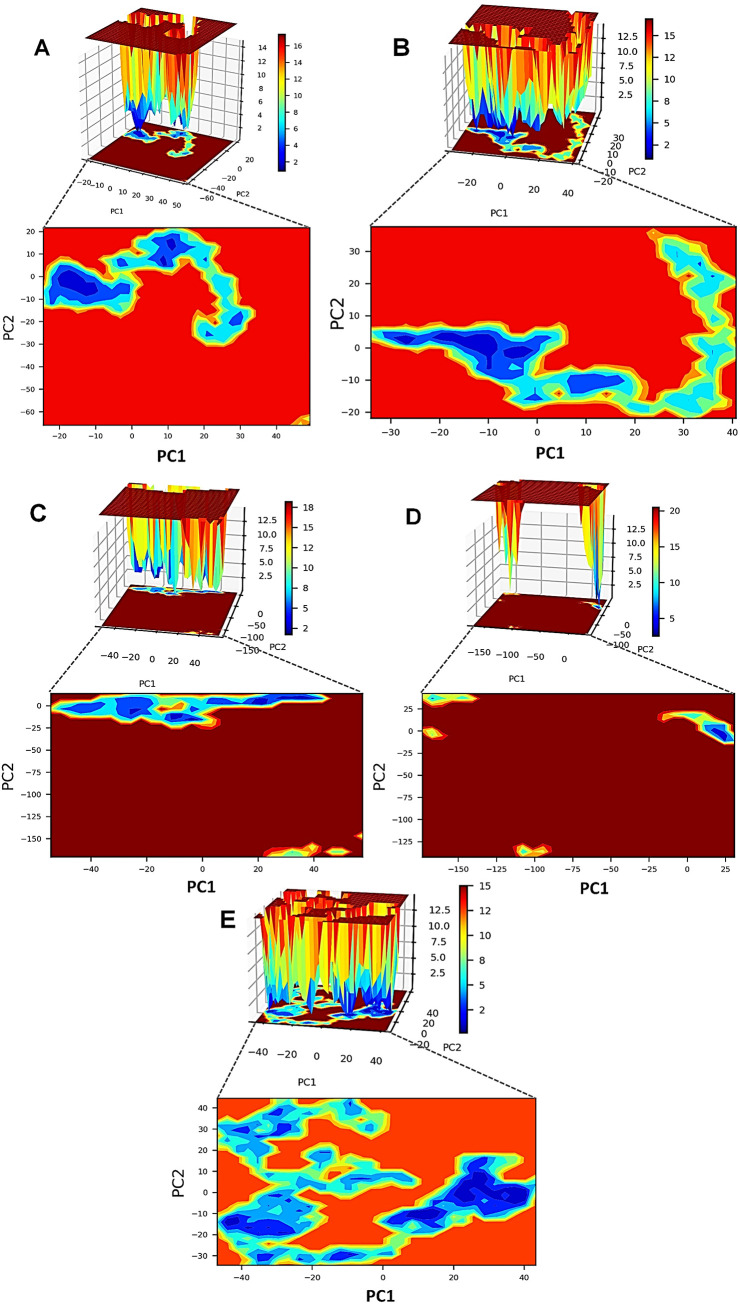
Gibb’s free energy landscape. (A) TLR1-vaccine complex. (B) TLR2-vaccine complex. (C) TLR3-vaccine complex. (D) TLR4-vaccine complex. (E) TLR81-vaccine complex.

**(vi) Principal-component analysis (PCA) and Gibb’s free energy analysis.** An essential dynamics analysis (PCA) was performed to understand the dynamics of each TLR-vaccine complex. The independent motions of the TLR and vaccine chains were observed in the PCA. The stability of TLR-vaccine complexes can be studied through Gibb’s free energy landscape (Gibb’s FEL) analysis. Two principal components (PC1 and PC2) were used to calculate Gibb’s free energy. The stability of the corresponding conformation and the system lower the Gibb’s free energy value. The results showed that the TLR8-vaccine complex has more of the lowest energy basins than other TLR-vaccine complexes. For the TLR8-vaccine complex, the energy range was −50 to 50 kJ mol^−1^ for PC1 and −35 to 45 kJ mol^−1^ for PC2 (Fig. 10). TLR2 and TLR1 vaccine complexes also showed the existence of lower energy basins comparable to the TLR8-vaccine complex. For the TLR2-vaccine complex, the energy range was −35 to 40 kJ mol^−1^ for PC1 and −20 to 40 kJ mol^−1^ for PC2, suggesting better system stability. Similarly, for the TLR1-vaccine complex, the energy range for PC1 was −20 to 50 kJ mol^−1^ and −60 to 20 kJ mol^−1^ for PC2, which suggests better system stability. For the TLR3-vaccine complex, the lowest energy conformations were limited to the narrow range of energy basins −50 to 40 kJ mol^−1^ on PC1 and −25 to around 10 kJ mol^−1^ on PC2. In the TLR4-vaccine complex, too few low-energy conformations were limited to the energy basin range −25 to 25 kJ mol^−1^ on PC1 and −25 to 25 kJ mol^−1^ on PC2.

**(vii) Dynamic cross-correlation (DCC) analysis.** DCC analysis was performed to analyze the time-correlated information of interchain and intrachain residue-to-residue contacts and motions. Here, the dynamic cross-correlation matrix (DCCM) (39) gave comprehensive residue information through the visual pattern. The results indicated each complex’s independent motions in the TLR and vaccine chains during the MD simulation. Fig. S8 at https://drive.google.com/file/d/1xprLNGfmlJjYNR_X_MdCnhzfknwhpYvO/view?usp=drivesdk shows the DCCM matrix for each TLR-vaccine complex. The color gradient in the figure ranges from blue (negative correlation) to red (positive correlation), corresponding to the correlation coefficients −1 and 1, respectively. The lighter shades of these colors indicate weaker correlation, (i.e., light blue corresponds to a correlation coefficient of −0.5, and the light red color corresponds to a correlation coefficient of 0.5). The white color indicates no correlation, corresponding to a correlation coefficient of 0. The results of the DCC analysis showed a strong negative correlation between TLR3 and the vaccine chain. Only a few residues from the TLR3 chain ranging from 497 to 597 (1,200 to 1,300 in a plot) showed a positive correlation with the vaccine chain, while it also showed a weaker intrachain positive correlation within a vaccine chain showing random motions in the vaccine chain. In the case of the TLR1-vaccine complex, the residues around 180 to 280 (700 to 800 in a plot) from the vaccine chain showed a weak correlation with the TLR1 chain. However, the intrachain correlation was better than the TLR3-vaccine complex. A slightly stronger positive correlation was observed with interchain contacts and motions of initial residues (i.e., residues 1 to 100 [800 to 900 in a plot] in the case of the TLR8-vaccine complex). In the TLR2-vaccine complex, almost all the residues of the vaccine chain showed a weak to strong positive correlation, signifying uniform contacts and motion in TLR2 and the vaccine chain. The strongest positive correlation was observed between vaccine chain residues and TLR8 chain residues spanning across all TLR8 and vaccine chain residues. Further, the intrachain correlation within the vaccine chain residues in the TLR8-vaccine complex was also better than in other TLR-vaccine complexes.

**(viii) Definition of the secondary structure of proteins (DSSP) analysis.** DSSP analysis provides insights into secondary structural changes during MD simulation in protein structures under study. The results showed that the secondary structures of all TLR chains almost remained unaltered concerning the coil, beta sheets, helices, and turns with minor transitions (Fig. S9 at https://drive.google.com/file/d/1xprLNGfmlJjYNR_X_MdCnhzfknwhpYvO/view?usp=drivesdk). The vaccine structure, where most of the structure is in coil, remained stable throughout the simulation.

**(ix) MM-PBSA calculation.** The trajectories extracted at 500 ps between 75- and 100-ns simulation periods were subjected to MM-PBSA calculation. Various binding interaction energies, such as van der Waal, electrostatic, polar solvation, Solvent Accessible Surface Area (SASA), and binding energy (Δ*G*_binding_), were estimated for TLR and the vaccine chain (36). The results of MM-PBSA calculations are given in Table 5. The results showed that the TLR4-vaccine complex has the most favorable binding energy of −1,019.36 kJ mol^−1^. The TLR3 and TLR8 complexes also have lower binding energies of −608.91 and −411.27 kJ mol^−1^, respectively. The TLR1 and TLR2 complexes have slightly higher binding energies, and the TLR2 complex was found to have the least favorable binding energy of −112.26 kJ mol^−1.^

**TABLE 5:** Results of MM-PBSA calculations

Complex with vaccine	van der Waal energy (kJ mol^−1^)^*a*^	Electrostatic energy (kJ mol^−1^)^*a*^	Polar solvation energy (kJ mol^−1^)^*a*^	Solvent accessible surface area energy (kJ mol^−1^)^*a*^	Binding energy (Δ*G*_binding_) (kJ mol^−1^)^*a*^
TLR1	−458.376 (4.144)	−399.930 (8.106)	649.301 (21.263)	−55.572 (0.695)	−260.148 (26.303)
TLR2	−171.729 (6.607)	−960.037 (6.464)	1,072.179 (24.463)	−31.865 (0.830)	−112.261 (59.118)
TLR3	−744.843 (8.707)	−1,636.055 (10.980)	1,877.355 (36.178)	−105.380 (1.050)	−608.910 (27.785)
TLR4	−328.337 (4.942)	−1,816.104 (29.438)	1,196.225 (26.737)	−48.631 (4.964)	−1,019.362 (51.893)
TLR8	−240.552 (3.343)	−789.116 (7.786)	652.763 (22.187)	−32.266 (0.412)	−411.275 (18.889)

a Standard deviations are given in parentheses.

### Immune simulation analyses.

**FIG 11:**
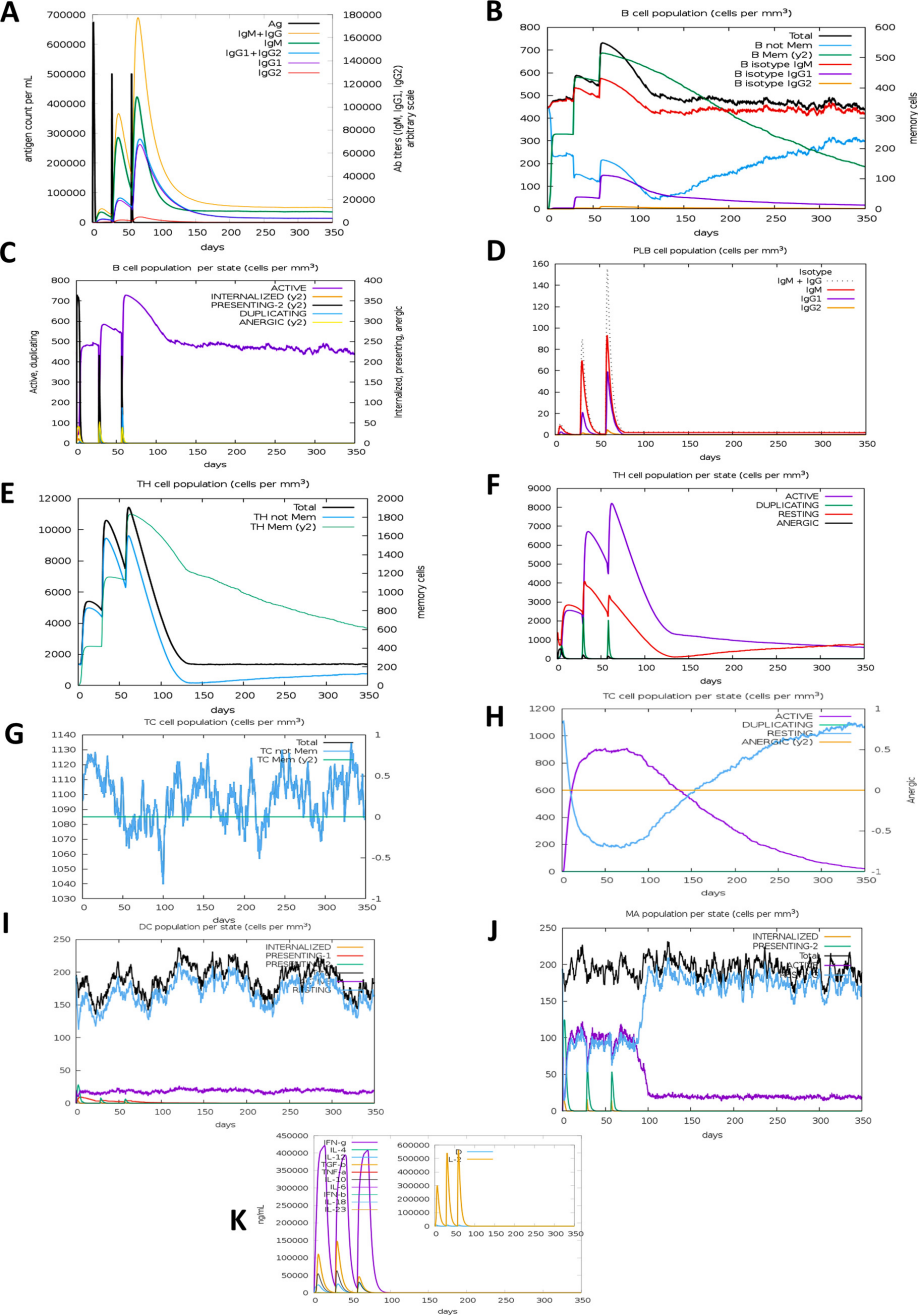
C-ImmSimm representation of the immune stimulation of the best-predicted vaccine, EV. (A) The immunoglobulin and immunocomplex response to the EV vaccine inoculations (lines colored in black) and the subclasses are indicated by colored lines. (B) Increase in the B-cell population throughout the three injections. (C) Inclination of the B-cell population per state throughout vaccination. (D) Rise in the plasma B-cell (PLB) population over the course of the injections. (E) Enhancement of the helper T-cell (TH) population throughout the three injections. (F) Increase in the helper T-cell population per state throughout vaccination. (G) Augmentation in the regulatory T lymphocyte (TC) throughout the three injections. (H) Rise in the cytotoxic T lymphocyte population throughout the injections. (I) Increment in the active cytotoxic T lymphocyte population per state over the course of the three injections; DC, dendritic cell. (J) Rise in the active dendritic cell population per state throughout the three injections; MP, macrophages. (K) Augmentation in the macrophage population per state over the course of the injections. (L) Increase in the concentrations of different types of cytokines throughout the three injections.

Immune stimulation of the EBV vaccine was performed in the C-ImmSimm server, which predicts if the epitopes might generate adequate adaptive immunity. Additionally, this analysis can determine the generation of immune interactions of the epitopes with their specific targets (40). From the immune simulation study, it was quite clear that the EBV vaccine has the potential to produce a typical immune response that is coherent with the natural and typical immune system. The vaccine was predicted to significantly induce primary immune responses after each of the three injections incrementally (Fig. 11A). Moreover, the secondary immune response was stimulated, and the primary immune response gradually increased after each dose.

Furthermore, successive increments in the concentrations of active B cells (Fig. 11B and C), plasma B cells (Fig. 11D), helper T cells (Fig. 11E and F), and cytotoxic T cells (Fig. 11G and H) were found, which suggests the generation of a highly potent immune response and immune memory as well as high clearance of the antigen in the host. Simultaneously, the stimulation of helper T cells pointed to the superior adaptive immunity that the vaccine provides (37, 41), while the increase in dendritic cells and macrophages pointed to excellent antigen presentation by the antigen-presenting cells (APCs) (Fig. 11I and J). An impressive range of various types of cytokines that are crucial for generating an immune response while defending the body from viruses, such as interferon-γ (IFN-γ), interleukin-23 (IL-23), IL-10, and IFN-β, could be produced by the vaccine (42, 43) (Fig. 11K). In addition, the study showed a negligible Simpson index (*D*), which drives conclusions toward the immune response of EBV virus being diverse (44). To summarize, the immune simulation studies predicted a range of promising abilities of the vaccine, including generating a large amount of immunoglobulins, APCs, cytokines, and active B cells and T cells, suggesting that the polyvalent EBV vaccine can induce excellent immunogenic responses after administration within the host.

### Codon adaptation, *in silico* cloning, and analysis of the vaccine mRNA structure.

**FIG 12:**
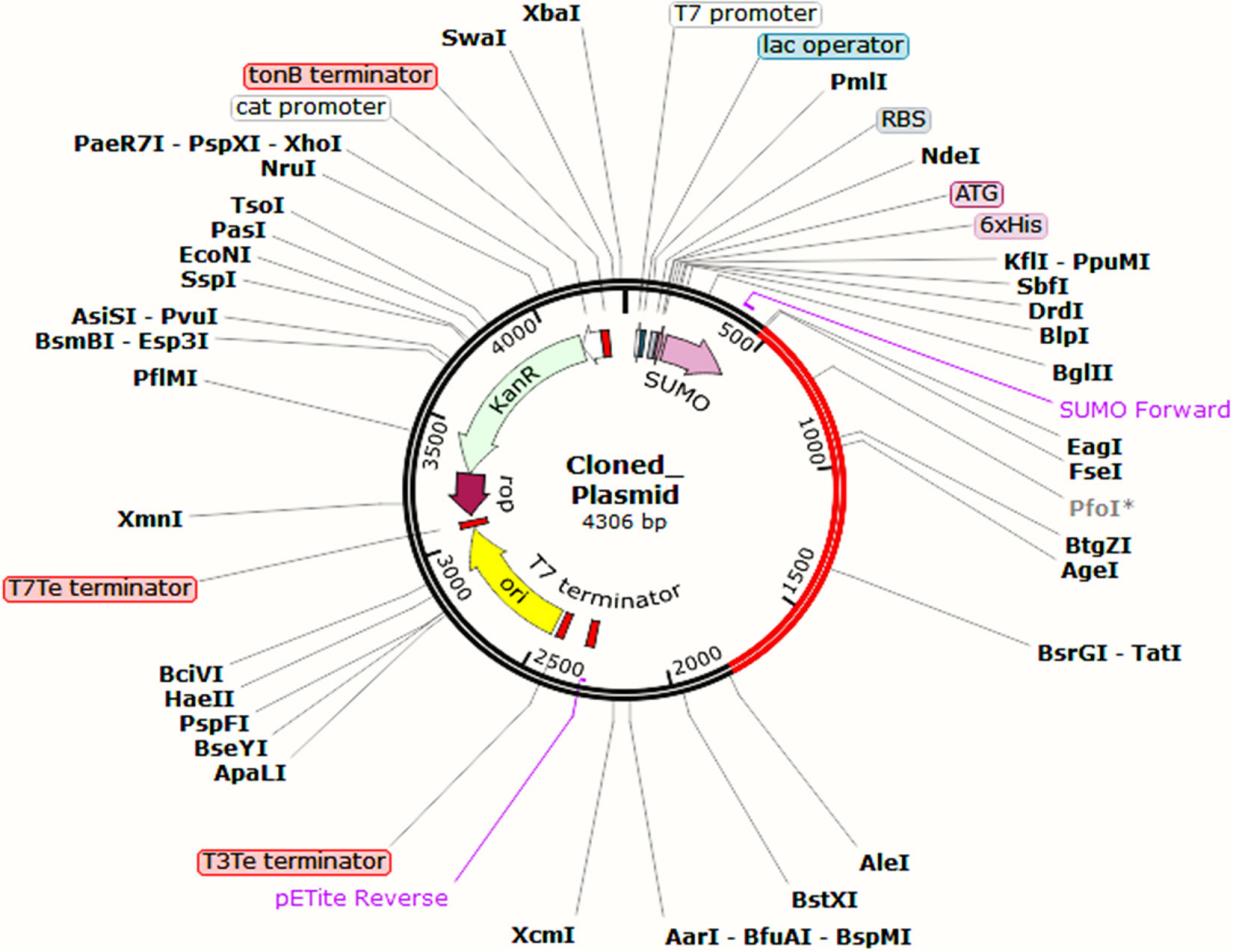
The recombinant plasmid was designed for mass production of the EV vaccine. Here, the red-colored portion depicts the designed EV vaccine construct.

From the codon adaptation and *in silico* cloning experiments, it was seen that the codon adaptation index (CAI) value of EV was more than the threshold, indicating a good result (0.89), ensuring an enriched presence within the DNA sequences and being the most likely codons to be used in cellular machinery of the target organism (E. coli strain K-12; Fig. S10 at https://drive.google.com/file/d/1xprLNGfmlJjYNR_X_MdCnhzfknwhpYvO/view?usp=drivesdk). The GC content of the improved sequence was 50.53%, which falls under the optimum score range of 30 to 70%. Following codon adaptation, the predicted DNA sequence of EBV was inserted into the pETite vector plasmid between the EaeI and StyI restriction sites, and due to the presence of sequences of the small ubiquitin-like modifier (SUMO) tag and 6× His tag in the plasmid, the vaccine protein was expected to be purified easily during downstream processing. The newly constructed cloned vector plasmid was designated “Cloned_Plasmid” (Fig. 12).

After that, the mRNA structure of the vaccine construct was predicted by the Mfold and RNAfold servers, and the minimum free energy score was determined (Fig. S11 at https://drive.google.com/file/d/1xprLNGfmlJjYNR_X_MdCnhzfknwhpYvO/view?usp=drivesdk). The Mfold server generated a minimum free energy score of −563.40 kcal/mol, which agreed with the prediction of RNAfold (−560.58 kcal/mol). As lower minimal free energy conforms to better mRNA stability, the predicted EBV vaccine may be stable after expression *in vivo* (45). Overall, the methods used in this study indicate the EBV vaccine as a potential countermeasure to combat EBV infection, which promises to be affordable and inexpensive. However, further *in vivo* and *in vitro* research studies are necessary to strengthen the outcomes of this study.

## DISCUSSION

Epstein-Barr virus (EBV), a gamma-1 herpesvirus, is usually carried as a lifelong persistent asymptomatic infection by the majority of human populations around the world. However, this seemingly unharmful viral agent is etiologically linked to several malignancies, including two premalignant lymphoproliferative diseases and nine distinct human tumors. This virus is ubiquitously distributed in the environment, and most of the world’s population gets infected with EBV at least once in their lifetime. EBV has been a major concern for decades, has a huge global impact, and is responsible for some 200,000 new cases of cancer arising worldwide each year (46). In recent years, many achievements in fundamental virology, vaccine technology, and synthetic biology have brought new opportunities for vaccine development against EBV. The lack of an approved effective vaccination against the virus has seemed extremely virulent, affecting millions worldwide. Because prevention is better than a cure, it is well established that vaccination is an excellent measure and a good alternative to treatments, providing immunity against different pathogenic organisms. There are different types of vaccines, such as peptide, conjugated, subunit, and DNA vaccines, which can be used to provoke the immune response. However, in this postgenomic and proteomic era, researchers prefer peptide or subunit vaccines over the whole pathogenic agent due to the availability and accessibility of huge data sets for different pathogens that can be systematically analyzed through different computational tools (47). This approach enables designing a vaccine that can achieve maximum effectiveness across different segments of the human population while reducing the chances of any adverse reaction.

In this immunoinformatics approach to designing a vaccine for EBV, three of the glycoproteins responsible for enhancing the virulence of the virus were targeted to design a blueprint of an epitope-based polyvalent vaccine against the two different strains belonging to EBV type 1 and type 2. Glycoproteins, present on the surface of viruses and virus-infected cells, have typically been primary candidates for developing vaccines to prevent infection and/or disease (48) because these surface antigens face the first encounter with the immune cells of the host. This study leverages a more nonconventional and swift methodology of computational biology for designing a multiepitope subunit vaccine to prohibit the infection of the virus using the vast genomic data available. Because immunoinformatics approaches have shown effective outcomes in designing vaccines, this study aimed to design a vaccine against EBV to reduce the global burden of multiple malignancies caused by the virus.

The immunoinformatics approach aims to eliminate limitations of finances and applications to present a viable solution for a novel vaccine. This approach demonstrates a blueprint of an epitope-based multivalent vaccine targeting the key virulent proteins of the virus, glycoproteins gB, gH, and gM. These target proteins were selected for this experiment, as in the literature found in the NCBI database. Using proteins of the EBV strain AG876 as the model, T-cell and B-cell epitopes were investigated. Various immunogenic filters were used to screen the cytotoxic T-lymphocyte (CTL) and HTL epitopes before inclusion in the vaccine construct. As the final selection for the construction of the vaccine, criteria, such as high antigenicity and nonallergenic, nontoxic, and nonhomolog properties to the human proteome, were fully ensured using stipulated thresholds. The HTL epitopes capable of inducing one or more cytokines in the human body were chosen to enable activation of the host’s adaptive immune system.

The EBV virus is not only a key agent in IM but also an active inducer of different cancers (49). This virus was the first tumor virus to be discovered and is associated with many human cancers originating from epithelial cells, lymphocytes, and mesenchymal cells in both immunocompetent and immunocompromised hosts (50). EBV infects and persists in certain white blood cells called B lymphocytes or B cells in the body. The T cells (i.e., CTLs and HTLs) are very important to generate a productive immunoreaction to protect the host from viral infection. However, the vaccine targeting CTL responses is less effective than those targeting CTLs and HTLs (51). In addition, these multiepitope vaccines have advantages over traditional and single-epitope vaccines due to the following unique features: (i) multiple MHC class T-cell receptors (TCRs) can recognize self and class II epitopes from various T-cell subsets, (ii) overlapping CTL, HTL, and B-cell epitopes can activate both humoral and cellular immune responses simultaneously, (iii) linking an adjuvant to the vaccine ensures a long-lasting, robust immune response with enhanced immunogenicity, (iv) and *in vitro* antigen expression complications as well as the difficulty of culturing the pathogens can also be avoided (52).

To ensure an enhanced immune response, the T-cell epitopes capable of binding to many MHC class I and class II molecules were selected using the IEDB prediction server, which rendered the prediction of the epitopes with a low percentile rank (<1.00), ensuring a higher affinity of the epitopes. The B-cell surface membrane receptors (BCRs) can directly recognize the solvent-exposed antigenic epitopes and induce the formation of antibodies specific to those epitopes (53). The linear or continuous B-cell epitopes were identified with the IEDB prediction server. The predicted Linear B-cell lymphocyte (LBL) epitopes with good scores were considered for further analyses. After the initial screening, 83 of the T-cell and B-cell epitopes were selected according to their corresponding scores, which were subsequently put through rounds of rigorous screening to identify the best epitope candidates displaying highly antigenic, nonallergenic, nontoxic, nonhomolog to the human proteome, and full conservancy properties across strains.

The cytokine-inducing ability of the HTL epitopes was also conducted to determine IFN-γ-, IL-4-, and IL-10-inducing properties of the HTL epitopes, respectively. Human beta-defensin-3 (hBd-3) was used as an adjuvant for the robust immunogenicity of the vaccine. PADRE sequence was also used as a strong immunity inducer (54). EAAAK, AAY, GPGPG, and KK linkers were used to link up the epitopes at their appropriate positions. EAAAK prevents vaccine degeneration when incorporated at the vaccine start or terminus. The use of adjuvants and linkers stabilized the vaccine construct and enriched the vaccine’s antigenicity, immunogenicity, and longevity profile.

The EBV vaccine demonstrates satisfying stability due to the high pI value of 9.53, conclusive of a basic range. The vaccine attains a half-life of 30 h in mammalian reticulocytes *in vitro* and more than 10 h in a prokaryotic E. coli culture system. The results indicate an unfaltering and constructive mass production possibility of the vaccine in the E. coli cell culture system, the most used system for mass production of recombinant proteins, ensuring the most stable propagation of recombinant proteins. Furthermore, the GRAVY value being −0.250 indicates the hydrophilic nature of the vaccine, deeming it more soluble in water. The other biophysical analysis results, such as the 77,100 M^−1^ cm^−1^ extinction coefficient measured at 280 nm in water, also indicate the vaccine’s solubility. The EBV vaccine also displayed high solubility predictions in SolPro and Protein-sol servers, acquiring solubility scores of 0.543 and 0.899.

The Ramachandran plot presented with a Z-score generated by the ProSA-web server during the secondary structure analysis of the EBV vaccine protein and the refined structure generated in the protein validation study suggests that the vaccine has quite an adequate structure and should be a satisfactorily effective vaccine. The Z-score of the vaccine is −8.5, which resides within the range of all experimentally proven X-ray crystal structures of proteins from the Protein Data Bank (PDB). Interestingly, the maximum number of amino acids was present in the best spots. Moreover, the vaccine protein generated five pairs of amino acids (i.e., Ser-27 and Ala-91, Ala-142 and Val-164, Ser-251 and Ala-271, Ala-298 and Val-316, and Ser-501 and Glu-504) with bond energy less than 2.2 kcal/mol, which was the selection criteria to determine the amino acids for mutation into cysteine residues.

A satisfactory result was seen in conformational B-cell epitope prediction of the vaccine, demonstrating seven potential regions capable of acting as conformational B-cell epitopes to the antibodies, with quite a good score ranging from 0.71 to 0.951 while covering a total of 291 amino acid residues. Docking of the EBV vaccine protein with different TLRs showed high binding affinity across all targets when docked using the ClusPro 2.0 and PatchDock servers. The high affinity with docking complexes simulates the genuine EBV vaccine’s epitopes binding with TLRs during immune responses.

In MD simulation studies, the higher RMSD in the TLR2 chain with concurrent slightly higher RMSD in the bound vaccine chain indicates a slightly unstable system. The lower magnitude of RMSD for TLR1, TLR3, and TLR4 suggests reasonable system stability. The vaccine chain bound to TLR1 is also indicative of system stability. The RMSD in TLR8 was most stable, with reasonably stable RMSD in the bound vaccine chain. The RMSD analysis revealed the favorable interactions between TLR chains, namely, TLR1, TLR3, TLR4, and TLR8, with the vaccine chain.

The RMSF in the TLR8 chain and corresponding vaccine chain suggests a large magnitude of fluctuations in RMSF. The higher magnitude of RMSF in this complex may be partly due to more interchain residue-to-residue contacts in this complex. A slightly higher magnitude of fluctuations in TLR3 and TLR4 chains with a concurrent slightly higher magnitude of RMSF in the corresponding vaccine chain suggests more frequent interchain residue-to-residue contacts. In TLR1 and TLR2 complexes, lower magnitude of RMSF suggests stability of the system and corresponding TLR chains.

The complete Rg analysis points out the least rotation of the TLR1 chain and the corresponding vaccine chain around its center of mass. This complex may be the most stable in terms of Rg evaluation. There is a larger magnitude of total Rg in the TLR3 and vaccine chains. The most stable Rg was found in the TLR8 chain, signifying its better stability along with the bound vaccine chain. The TLR2 and TLR4, along with the bound vaccine chain with slightly lower Rg, are also reasonably stable.

A maximum number of around 30 hydrogen bonds were formed after around a 50-ns simulation period in the TLR3-vaccine complex, suggesting better stability and vaccine chain affinity to the TLR3 chain. More than 20 hydrogen bonds in TLR4 and TLR8 vaccine complexes also indicate the better affinity of the vaccine chain to these TLRs. Around 10 hydrogen bonds formed in TLR1 and TLR2 complexes with the vaccine chain may have slightly lower vaccine chain affinity to these TLRs than other TLR complexes. However, neither particular residue from TLR nor vaccine chains form a stable hydrogen bond in these complexes.

The contact analysis through contact maps revealed more interchain residue-to-residue contacts in the TLR3-vaccine complex. In the case of the TLR2 and TLR8 vaccine complexes, three specific regions on the contact map showed the interchain contact at three different domains of proteins. The TLR2-vaccine complex showed fewer contacts and suggested a weaker association between the TLR and vaccine chains. In the TLR4-vaccine complex, the interchain contacts were limited to closely associated residues, while in the TLR8-vaccine complex, diverse residues from the vaccine chain established contact with the TLR chain.

Gibb’s free energy evaluation identified the most favorable, lowest energy conformations in the TLR8-vaccine complex. The ground state-free energy was also lower than other TLR complexes with the vaccine. TLR2 and TLR1 complexes with vaccines also showed favorable free energy basins where large numbers of lowest energy conformations belonging to a large cluster were formed. The free energy state for TLR3 and TLR4 was found slightly unfavorable, as smaller numbers of conformations lie in the lowest energy states.

The DCC analysis pointed out many positively correlated contacts between TLR4-vaccine residues and TLR2-vaccine residues. The TLR4 and TLR2 complexes with the vaccine also have many strong negatively correlated contacts. The TLR1, TLR3, and TLR8 complexes with the vaccine have intermediate positively and negatively correlated residue contacts during simulation. Thus, the DCC analysis suggests a better association between the TLR4-vaccine complex and the TLR2-vaccine complex.

The DSSP analysis revealed no major secondary structural changes in the TLR chain. However, the secondary structural changes in loop regions of the vaccine chain are evident in all the complexes. The TLR chains are rugged to secondary structural changes.

The MM-PBSA analysis revealed that the TLR3-vaccine complex has the lowest van der Waal energy, while the TLR4-vaccine complex has the lowest electrostatic energy. However, the greater extent of polar solvation energy for the TLR3-vaccine complex results in its slightly higher binding energy than the TLR4-vaccine complex. The contribution of solvent-accessible surface area energy is almost the same for all the TLR-vaccine complexes, except the TLR3-vaccine complex, which is 2-fold lower than other complexes. Higher van der Wall energy and concomitant higher polar solvation energy are responsible for slightly higher binding energy for the TLR2-vaccine complex than for other TLR-vaccine complexes. The short but computationally expensive MM-PBSA calculations highlight the stability and favorable interactions in vaccine TLR4 and TLR3 complexes.

The mRNA of the vaccine was enhanced with the help of the Java Codon Adaptation tool, choosing the E. coli strain K-12 as the cell culture system to predict the translation efficiency of the EBV vaccine (55). A range of expected numerical results was found; the codon adaptation index (CAI) value was 0.89, and the GC content was 50.53%. These outcomes were considered satisfactory as any CAI value over 0.80 and a GC content from 30 to 70% represent good scores. Afterward, during codon adaptation in the pETite plasmid, the restriction enzymes were made to cut the N and C termini, determined to be EaeI and StyI, respectively. The presence of SUMO and 6× His tags in the cloned plasmid will enable posttranslational vaccine purification. Moreover, the Mfold and RNAfold servers predicted the secondary structure of the EBV mRNA, generating the minimum free energy scores of −563.40 kcal/mol and −560.58 kcal/mol, respectively. Such lower scores indicate higher vaccine stability in the body, which is highly expected during vaccine construction.

This study provides a means for developing subunit vaccines against EBV infection that could prevent many important diseases. Such computational meta-analysis integrated with dynamics has been reported promising for vaccine designing against the virus. Thus, it has sufficiently increased the scope and precision of this study (56). Our results will facilitate efficient experimental efforts, as the three different envelope glycoproteins of EBV strains B95-8 and AG876 (i.e., gB, gH, and gM) could be used for construction of the vaccine by conjugating the epitopes (CTL, HTL, and LBL epitopes), whereas previously only single epitope-based peptide vaccines were proposed to fight against EBV infection. However, further wet lab-based studies on the designed vaccine are required to justify the intriguing results and confirm the vaccine’s safety, efficacy, and potentiality.

### Conclusion.

EBV is a virulent oncovirus associated with several malignancies and different cancers, including nasopharyngeal cancer, lymphoma, and some cases of stomach cancer. Millions of people get infected with this virus every year, and many patients require hospitalization. Although current studies have successfully developed vaccines to prevent this virus over the past few decades, no highly efficient vaccine is yet available on the market. In this study, a possible epitope-based polyvalent vaccine was designed against EBV (EBV-1 and EBV-2) using the tools of immunoinformatics and *in silico* biology. The vaccines occupied fully conserved T-cell and B-cell epitopes; hence, they might generate an effective immune response against the two selected viruses.

Moreover, the high antigenicity, nonallergenicity, nontoxicity, conversancy, cytokine-inducing ability (for MHC class II epitopes), and nonhomology (to the human proteome) were also considered the criteria to select the most promising epitopes for final vaccine construction. Consequently, the vaccine is expected to generate a very good immunogenic response without exacerbating any unwanted reaction within the body. The experiments predicted that the designed polyvalent vaccine should be safe, effective, and responsive to administration. However, since all the predictions were made using only *in silico* methods, further wet lab-based studies are required to confirm this study’s outcomes. With high-cost requirements and different limitations of developing the live, attenuated, or inactivated vaccine preparation for such a highly infectious agent, peptide-based vaccine candidates like the one designed in this study might be a relatively less expensive and more effective option to successfully reach the entire world to combat the malignancies related to EBV infections.

## MATERIALS AND METHODS

A flowchart of the high-throughput immunoinformatics approaches utilized for vaccine design is illustrated step by step in Fig. 1.

### Strain selection and retrieval of the protein sequences.

According to the NCBI (https://www.ncbi.nlm.nih.gov/) database, two unique strains of EBV, strain B95-8 and strain AG876 belonging to type 1 and type 2, respectively, were recognized, and three distinct envelope glycoproteins of the virus (gB, gH, and gM) were selected. The sequences of the target proteins of the selected strains were then extracted from the UniProt database (https://www.uniprot.org) in FASTA format.

### Biophysical analyses and prediction of the *N*-glycosylation sites of targeted proteins.

Antigenicity refers to the ability of a biomolecule to be specifically detected by the antibodies that have been generated due to the immune response against the antigen of that specific biomolecule (57–59). Using the online antigenicity prediction tool VaxiJen v2.0 (http://www.ddg-pharmfac.net/vaxijen/VaxiJen/VaxiJen.html), a range of the particular protein sequences retrieved from the two virulent strains of EBV were analyzed, keeping the prediction accuracy parameter at 0.4. This server is widely accepted because it uses the transformation method of auto cross-covariance (ACC) to predict the antigenicity of query proteins with an accuracy of around 70% to 89% (60). The ProtParam tool of the ExPASy server (https://web.expasy.org/protparam/) was used to determine the numerous physicochemical properties of the selected proteins. Our selected proteins’ different physiochemical parameters, including the number of amino acids, molecular weight, total atoms, theoretical pI, instability index, extinction coefficient, half-life, and grand average of hydropathicity (GRAVY), were computationally analyzed through the server (61). Following that, the protein sequences were subjected to prediction of *N*-glycosylation sites using the NetNGlyc-1.0 server (https://services.healthtech.dtu.dk/service.php?NetNGlyc-1.0). The NetNglyc server analyzes the sequence context of Asn-Xaa-Ser/Thr sequons to predict *N*-glycosylation sites in human proteins using artificial neural networks. Any potential greater than 0.5 indicates the presence of a predicted glycosylated site.

### T-cell and B-cell epitope prediction.

For a multipeptide subunit vaccine to effectively stimulate an immune response in the host, the presence of cytotoxic T-cell, helper T-cell, and B-cell epitopes is necessary. Epitope discovery for vaccine construction has been increasingly reliant on different immunoinformatic analytical tools because of the option of accessing various depositories of data relevant to the specified immune reaction or specific pathogens. The T-cell and B-cell epitopes of the selected protein sequences were predicted using the online epitope prediction server Immune Epitope Database (IEDB; https://www.iedb.org/), keeping all the default parameters during prediction (62, 63). The IEDB tool utilizes validated and benchmarked methods to predict MHC molecule binding, antigen processing, TCR recognition, and B-cell epitope prediction. The recommended NetMHCpan EL 4.0 prediction method (http://tools.iedb.org/mhci/) was utilized for the prediction of MHC class I-restricted CD8^+^ cytotoxic T-lymphocyte (CTL) epitopes of the selected sequences for some common human leukocyte antigen (HLA) alleles (i.e., HLA A_01:01, HLA A_02:01, HLA A_02:06, HLA A_03:01, HLA A_11-01, and HLA A_29:02), keeping the length of the epitopes at 9 (9-mer epitopes). The default prediction method for selecting the server was “IEDB recommended,” which utilizes the best technique for MHC molecule identification based on the availability of predictors and observes the predicted performance for the specific allele.

Similarly, we determined the MHC class II-restricted CD4^+^ helper T-lymphocyte (HTL) epitopes for some common HLA alleles (i.e., DRB1_03:01, DRB1_04:01, DRB1_15:01, DRB5_01:01, DRB4_01:01, and DRB3_01:01) using the IEDB recommended 2.22 prediction method (http://tools.iedb.org/mhcii/) and keeping the length of the epitopes at 15 (15-mer epitopes). The IEDB recommended method uses the Consensus method combining four approaches, NN-align, SMM-align, CombLib, and Sturniolo, if any corresponding predictor is available for the MHC molecule. In the absence of these options, the NetMHCIIpan is utilized. Based on the rankings provided by these prediction tools, the top 10 MHC class I and II epitopes found to be common for all the mentioned HLA alleles were chosen for further analyses. The default parameters were retained during T-cell epitope prediction.

The selected proteins’ LBL were predicted using the BepiPred linear epitope prediction method 2.0, retaining all the default parameters. Using a random forest algorithm trained on epitope and nonepitope amino acids obtained from crystal structures, the BepiPred-2.0 server predicted linear B-cell epitopes for our selected protein sequence. In the next step, a sequential prediction smoothing was conducted, and residues with scores greater than the threshold (default value of 0.5) were considered more probable to constitute epitopes (64). Based on this analysis, the top-scored LBL epitopes containing more than 10 amino acids were deemed potential candidates for further analysis.

### Antigenicity, allergenicity, toxicity, and transmembrane topology prediction.

Conservancy, antigenicity, allergenicity, toxicity, and transmembrane topology were predicted to identify the viability of the initially predicted epitopes for constructing the subunit potential vaccine. As an ideal vaccine, the vaccine’s components should be highly antigenic but nonallergenic and devoid of toxic reactions. The selected epitopes were run again through the VaxiJen v2.0 server for detection of antigenicity, keeping the prediction accuracy threshold at 0.4 (60). The selected epitopes’ allergenicity determination was assessed using two online tools, AllerTOP v2.0 (https://www.ddg-pharmfac.net/AllerTOP/) and AllergenFP v1.0 (http://ddg-pharmfac.net/AllergenFP/). Allergenicity is a property encoded within the sequences in a possibly concealed manner. Thus, alignment-based approaches cannot properly detect the allergenicity of the biomolecules.

Consequently, servers employing the alignment-independent auto cross-covariance (ACC) method, such as AllerTop and AllergenFP, were used here. Although both of the tools are based on auto cross-covariance (ACC) transformation of protein sequences into uniform equal-length vectors, results predicted by AllerTOP v2.0 were given priority due to the server having a slightly better accuracy of 88.7% than the AllergenFP server that has an accuracy of 87.9% (65, 66). The ToxinPred server (http://crdd.osdd.net/raghava/toxinpred/) was used for toxicity prediction of the selected epitopes, keeping all the default parameters default and utilizing the support vector machine (SVM) method. The SVM, a widely accepted machine learning technique for toxicity prediction, is estimated to differentiate the toxic and nontoxic epitopes (67) efficiently. Finally, the transmembrane topology of protein helices determinant, the TMHMM v2.0 server (http://www.cbs.dtu.dk/services/TMHMM/), was used, keeping all the default parameters to predict whether the epitopes were exposed inside or outside. With satisfying accuracy, the transmembrane topology prediction based on the hidden Markov model (HMM) (68) helped us determine the transmembrane topology of the screened epitopes.

### Cytokine-inducing capability determination of the HTL epitopes.

Helper T cells generate a range of cytokines, such as IFN-γ, IL-4, and IL-10, which activate other immune cells, including the cytotoxic T cells, B cells, and macrophages (40). Thus, it is crucial to assess the ability of the HTL epitopes to stimulate the production of these cytokines to induce an appropriate immune response by the vaccine. The ability of the HTL epitopes to induce IFN-γ, IL-4, and IL-10 were respectively predicted by IFNepitope (http://crdd.osdd.net/raghava/ifnepitope/), IL4pred (http://crdd.osdd.net/raghava/il4pred/), and IL10pred (http://crdd.osdd.net/raghava/IL-10pred/) servers. These servers are easy to operate and provide user-friendly tools, making them widely used to predict the cytokine-inducing ability of specified regions within query biomolecules (69–71). The Hybrid (Motif + SVM) prediction approach was used to predict the vaccine’s IFN-γ-inducing capability. This Hybrid prediction approach is highly precise in predicting the epitope-inducing capacity of IFN-γ (69). In addition, using the SVM method, the IL-4- and IL-10-inducing HTL epitope properties were determined using the servers IL4pred (https://webs.iiitd.edu.in/raghava/il4pred/index.php) and IL10pred (http://crdd.osdd.net/raghava/IL-10pred/), respectively (70, 71), retaining the default threshold values at 0.2 and −0.3, respectively.

### Conservancy and human homology determination of the epitopes.

The conservancy analysis in immunoinformatics studies is performed to determine the degree of epitope dispersion in a homologous protein collection (72). The “epitope conservancy analysis” module of the IEDB server (http://tools.iedb.org/conservancy/) was utilized for the conservancy analysis of the selected epitopes. On analysis, the epitopes exhibiting exclusive conservation across the selected strains were considered for vaccine construction to ensure the broad-spectrum activity of the polyvalent vaccine against the selected strains. It was essential to unleash any homology of the epitope to the human proteome. Homology of the human proteome epitopes was determined by the BLAST (BLASTP) module (https://blast.ncbi.nlm.nih.gov/Blast.cgi), where Homo sapiens (taxid: 9606) was used for comparison. After retaining all the other default parameters, an E value cutoff of 0.05 was set, and epitopes with no hits below this E value threshold were selected as nonhomologous pathogen peptides (73). Among all the initially selected epitopes, those found to be highly antigenic, nonallergenic, nontoxic, fully conserved, and nonhomologous to the human proteome were deemed the most promising and utilized in the construction of the vaccine.

### Population coverage and cluster analysis of the epitopes.

Population coverage analysis assesses the fraction of individuals within a population likely to respond to a particular set of epitopes possessing known MHC restrictions. The distribution of specific HLA alleles among various populations and ethnicities worldwide is a crucial step before constructing a multiepitope vaccine because the frequencies of expression of different HLA types greatly vary across different ethnicities (72). Thus, our selected epitopes should bind to the several alleles of HLA supertypes for maximum efficacy to ensure maximum population coverage. The population coverage of the best-selected epitopes across multiple HLA alleles over different regions of the world was analyzed with the IEDB population coverage tool (http://tools.iedb.org/population/) (72, 74), keeping all the default values of the webserver.

### Cluster analysis of the MHC alleles.

Cluster analysis of the MHC alleles aims to differentiate the MHC class I and class II alleles having identical specificities and demonstrate this relationship of the clusters of the alleles phylogenetically (75). MHCcluster 2.0 (http://www.cbs.dtu.dk/services/MHCcluster/) (76), a user-friendly online tool, was used to perform the cluster analysis of the MHC alleles of the selected protein epitopes. The output generates a static heat map and graphical tree-based visualizations of the functional relationship between the MHC variants and a dynamic TreeViewer interface that displays both the functional relationship and the individual binding specificities of the MHC molecules (76). Fifty thousand peptides were included, 100 bootstrap calculations were retained, and all the HLA supertype representatives (MHC class I) and HLA-DR representatives (MHC class II) were selected to perform the analysis.

### Multiepitope subunit vaccine design.

The “most promising” epitopes showed the best results in antigenicity, were nonallergenic and nontoxic, displayed nonhomology to the human proteome, and were highly conserved across the selected strains. These selected antigenic epitopes were scrutinized to assess their ability to induce cytokines, and those HTL epitopes capable of inducing at least one cytokine were selected for vaccine construction. After analysis, the most promising epitopes that met the specified criteria were selected to construct a polyvalent vaccine against EBV. The best-selected CTL, HTL, and LBL epitopes were conjugated by an adjuvant PADRE sequence and different linkers to construct the vaccine. An adjuvant is a vital element for vaccine construction, a substance that can substantially enhance the potential vaccine’s antigenicity, immunogenicity, stability, and longevity (77, 78). Human beta-defensin-3 was selected as the adjuvant sequence in this study. Human beta-defensins (hBds) are antimicrobial peptides produced by epithelial cells either constitutively (hBd-1) or after being induced by microbial products, inflammatory molecules, or epidermal growth factors (79–82). The chemotactic properties of hBD and major role in host defense, such as chemoattraction of immature dendritic cells, naive memory T cells, and monocytes to the site of infection, suggest a possible role of these peptides in mediating innate and adaptive host defense (83).

Similarly, the antibacterial activity and immunomodulatory function of hBd-3, the most recently discovered member of the host defense peptide family, is very significant in shaping the inflammatory responses in tissues and serving as a bridge between innate and innate adaptive defenses (84). Additionally, administration induces the activation of Toll-like receptors TLR-1, TLR-2, and TLR-4 inside the human body (77, 78). TLRs can recognize the difference between self and nonself by detecting highly conserved microbial patterns termed pathogen-associated molecular patterns (PAMPs) (23, 85). The adjuvant was putatively linked to the epitopes with the help of EAAAK linkers, followed by the PADRE sequence associated with the adjuvant and the epitopes. The PADRE sequence improves the capacity of the CTL by provoking the immune response produced by the vaccine (86, 87). Afterward, the epitopes CTL, HTL, and LBL were linked in a consecutive sequence with the help of the EAAAK, AAY, GPGPG, and KK (88, 89). In the case of a bifunctional fusion protein, the EAAAK linkers effectively separate the protein domains (90).

However, the flexible AAY linkers protect the proteins against degradation (91). The GPGPG linkers accelerate immune processing and presentation while preventing junctional epitope generation (92). Moreover, the KK linkers, also known as the bilysin linkers, preserve the independent immunological activities of the epitopes of a vaccine (93).

### Antigenicity and allergenicity tests to assess the physicochemical properties of the vaccine.

Higher antigenicity ensures that the host immune system recognizes the vaccine construct and elicits a reaction triggering the immune cells (71). Similarly, allergenicity tests ensure that the vaccine will not trigger any allergic reaction in the host. Additionally, an in-depth evaluation of various physicochemical factors is essential to ensure the safety and efficacy of the vaccine (94). VaxiJen v2.0 (http://www.ddg-pharmfac.net/vaxijen/VaxiJen/VaxiJen.html) was used to determine the antigenicity of the constructed vaccine, keeping the accuracy threshold at 0.4. Simultaneously, the allergenicity of the vaccine construct was determined using the AlgPred (http://crdd.osdd.net/raghava/algpred/) and AllerTop v2.0 (https://www.ddg-pharmfac.net/AllerTOP/) servers. The AlgPred server combines different antigenicity-determining methods and uses the similarity of any known protein region’s known epitope to predict potential allergenicity.

In contrast, AllerTop v2.0 uses MEME/MAST motif prediction to predict the allergenicity of the vaccine construct (95, 96). The physicochemical properties of the vaccines, such as pI value, half-life, and GRAVY value, were again determined by ProtParam (https://web.expasy.org/protparam/). Last, solubility of the vaccine construct was predicted using the SOLpro module of the SCRATCH protein predictor (http://scratch.proteomics.ics.uci.edu/), and the result was further verified using the Protein-Sol server (https://protein-sol.manchester.ac.uk/), retaining all the default parameters during prediction. A higher solubility factor ensures that the vaccine will be properly soluble once administered in the host; otherwise, even a very effective vaccine will form insoluble aggregates. The SolPro server generates solubility predictions based on the SVM method, while Protein-Sol uses a rapid method of deciding the results based on their sequence. Consequently, results from both of these servers are considered reliable in predicting the solubility of any protein sequences (38, 97).

### Secondary and tertiary structure prediction of the vaccine construct.

Assessing a vaccine construct’s secondary and tertiary structures is crucial because the protein function depends on its structural conformation. Followed by antigenicity and allergenicity tests, the secondary structure of the vaccine construct was determined by the online PRISPRED (http://bioinf.cs.ucl.ac.uk/psipred/) tool, keeping all the default parameters. PRISPRED is a simple tool for generating secondary structure and predicting the transmembrane topology, transmembrane helix, fold, and domain recognition of the given biomolecule (98, 99). Two-dimensional (2D) structure analysis was conducted using GOR IV, SOPMA, and SIMPA96 servers for comparison and a strong validation of the results. The tertiary structure of the vaccine construct was generated using the online RaptorX (http://raptorx.uchicago.edu/) tool, keeping all the default parameters. The RaptorX tool predicts structure and *P* values for the predicted structures of the query proteins by a template-based method. The *P* value represents the model quality of the predicted protein structure, and the lower the *P* value, the better the model quality it designates (100, 101).

### Three-dimensional structure refinement and validation of the vaccine construct.

Accuracy assessment of vaccine constructs generated through *in silico* approaches is a key factor, especially where experimental evidence is scarce. Building 3D models may not always ensure the accuracy level for biomedical applications as an experimental accuracy would have provided. By performing 3D structure refinement, it was possible to improve the accuracy of initially predicted structures and correct local errors while retaining the vaccine’s near-accurate native structure. Refinement of the 3D structures of the vaccine was conducted by the GalaxyRefine module of the GalaxyWEB server (http://galaxy.seoklab.org/). The server applies a CASP10-tested method for refinement and dynamics simulation to provide improved structures (102, 103). However, delivering accurate and consistent refinement of 3D vaccine models is still challenging, especially at high resolution (104). Hence, to validate the vaccine constructs, Ramachandran plots were generated with the PROCHECK (https://saves.mbi.ucla.edu/) server (105, 106). Plot analysis displayed the allowed and disallowed dihedral angles psi (ψ) and phi (ϕ) of the amino compositions, which were calculated considering the van der Waal radius of the side chain. Another online tool, the ProSA-web (https://prosa.services.came.sbg.ac.at/prosa.php), worked on different statistical methods to generate Z-score and was also applied for validation of the proteins. The Z-score designates the quality of the query protein structure. The latest PDB database provides a range of Z-scores of all experimentally determined protein chains, which helps determine any query protein’s better quality and higher consistency (107).

### Assessment of vaccine protein disulfide engineering.

Improving the stability of protein vaccines through different molecular interactions is an important part of designing vaccines (108). The disulfide engineering of the vaccine protein was conducted with the online tool Disulfide by Design 2 v12.2 (http://cptweb.cpt.wayne.edu/DbD2/), a server that predicts the possible sites within a protein structure more likely to undergo disulfide bond formation. The tool was developed to predict protein structure using computational approaches (109, 110), and the algorithm of this server uses a geometric model derived from the native disulfide bonds to accurately estimate the *χ*3 torsion angle based on the 5th Cβ-Cβ distance. Due to the wide range of native disulfide bonds, the Caf-Cβ-Sγ angle is allowed some tolerance in the DbD2 server. DbD2 estimates an energy value for each potential disulfide to facilitate the ranking process, and mutant PDB files may be generated for selected disulfides (108).

The intra-chain, and inter-chain disulfide bonds were predicted in this analysis. Mutation of the cysteine residues to form disulfide bonds is necessary, and for that to happen, the *χ*3 angle was kept at −87 or +97 ± 5°. The Cα-Cβ-Sγ angle was kept at its default value of 114.6 ± 10°. The amino acid pairs with less than 2.2 kcal/mol bond energy were selected. As 90% of the native disulfide bonds generally have an energy value of less than 2.2 kcal/mol (111), this value was selected as the threshold bond energy.

### Protein-protein docking analysis.

Docking provides a structural framework for drug design and fundamental investigation tools into protein interactions. Protein-protein docking predicts the structure of a complex from the constituent protein structures (112). In the protein-protein docking study, the vaccine construct was analyzed by docking against various TLRs (i.e., TLR-1 [PDB ID: 6NIH], TLR-2 [PDB ID: 3A7C], TLR-3 [PDB ID: 2A0Z], TLR-4 [PDB ID: 4G8A], and TLR-8 [PDB ID: 3W3M]). Various online docking tools were used to perform protein-protein docking analysis to improve prediction accuracy. First, docking was performed by ClusPro 2.0 (https://cluspro.bu.edu/login.php), a server that arranges the clusters of docked complexes in order of rank based on their lowest energy scores and their center. The ClusPro server uses the equation: *E* = 0.40*E*_rep_ + (−0.40*E*_att_) + 600*E*_elec_ + 1.00*E*_DARS_ to calculate the energy score (113, 114). The repulsions and attraction energies of van der Waals interactions are denoted by *E*_rep_ and *E*_attr_ in the equation, respectively, whereas *E*_elec_ denotes the electrostatic energy component. *E*_DARS_ represents the Decoys’ pairwise structure-based potential as the reference state (DARS) method. The lower energy score obtained from this equation represents a stronger binding affinity. The second round of docking was conducted by the PatchDock server (http://bioinfo3d.cs.tau.ac.il/PatchDock/), keeping all the parameters to default values (115). The specific algorithm tool analyzes the Connolly dot surface representations and the candidate compounds’ root means square deviation (RMSD) clustering scores. Another round of docking was performed using the FireDock server (https://bioinfo3d.cs.tau.ac.il/FireDock/) to refine and restore the obtained results (116). Like Clustpro, the FireDock server ranks the docked complexes based on their global energy score, whereas a lower energy score signifies better affinity. The final round of docking was performed using the HawkDock server (http://cadd.zju.edu.cn/hawkdock/).

Along with the docking, the molecular mechanics/generalized born surface area (MM-GBSA) study was also conducted, retaining all the default parameters (117–119). After successful docking, the TLRs docked with the vaccine construct were taken for visualization and the MD simulation study. The docked complexes were visualized by the Discovery Studio Visualizer (120).

### Conformational B-cell epitope prediction.

Humoral immunity is dependent on the antibody-producing plasma B cells that produce antibodies on encountering antigens. So, the vaccines must possess effective conformational B-cell epitopes to provide much stronger immunity. The IEDB ElliPro tool (http://tools.iedb.org/ellipro/) was used to determine conformational B-cell epitopes of the vaccine, keeping default parameter values. The tool predicts linear and discontinuous antibody epitopes based on the protein antigen’s 3D structure. For each predicted epitope, a protrusion index (PI) score is provided that dictates the percentage of the residues outside ellipsoids used to divide the protein structure into segments. Residues with larger scores dictate greater solvent accessibility (121).

### Molecular dynamics simulation and MM-PBSA calculations.

**(i) Molecular dynamics simulation.** The docked complexes of TLR1-TLR4 and TLR8 with vaccine were subjected to 100-ns MD simulations to study the stability and evolution of structural changes in respective TLRs and vaccines. Each system’s TLR and vaccine chains make more than 1,000 residues. MD simulations of such multichain systems were performed on the HPC cluster at Bioinformatics Resources and Applications Facility (BRAF), C-DAC, Pune, with a preinstalled Gromacs 2020.4 package (122).

The modeler 9.12 program modeled and filled the missing residues in TLRs (123). The input topologies for TLR and vaccine chains were obtained using CHARMM-36 force field parameters (124, 125). While preparing the systems for MD simulations, each TLR chain and vaccine chain system was placed in a dodecahedron unit cell, and the system was solvated with transferable intermolecular potential 3P (TIP3P) water molecules (126). Later, the system was neutralized with the addition of chloride counterions, as each system has been found to have a positive charge. Such neutralized systems were subjected to the energy minimization step with the steepest descent minimization algorithm until the threshold (*F*_max_ < 1,000 kJ mol^−1^ nm^−1^) was reached. Furthermore, the system was equilibrated at a constant volume and constant temperature of 300 K (NVT) and under constant volume and constant pressure (NPT) conditions. A modified Berendsen thermostat (127) was used to achieve NVT conditions, while Berendsenbarostat (128) was used to achieve NPT conditions. Each NVT and NPT equilibration was performed with a short 1-ns simulation.

Finally, complete five production phase simulations (i.e., TLR1-vaccine, TLR2-vaccine, TLR3-vaccine, TLR4-vaccine, and TLR8-vaccine) for a duration of 100 ns with 1-fs step size were performed on the equilibrated systems with the same thermostat and Parrinello-Rahman barostat (129). All covalent bonds were restrained with the LINCS algorithm during production-phase MD simulations (130). The long-range electrostatic interaction energies were measured with the particle mesh Ewald (PME) method (131) with the cutoff distance of 12 Å. After removing the periodic boundary conditions (PBCs), the MD trajectories were used in the post-MD simulation analysis. The molecular deviations in TLR-vaccine dynamics were investigated by analyzing root mean square deviations (RMSDs) in C-α atoms of each system’s TLR chain and vaccine chain (132). The residue-wise local alterations were analyzed through each system chain’s root mean square fluctuation (RMSF). The TLRs have a unique secondary structure, and the dynamics of folding compactness and the vaccine chain were analyzed through the radius of gyration (Rg). Hydrogen bonding, a nonbonded interaction important in the overall stability of the TLR-vaccine combination, was analyzed through the gmx hbond program. The number of hydrogen bonds between TLR and vaccine chains was plotted against simulation time.

In addition to these analyses, the residue-to-residue contacts analysis was analyzed through contact map analysis (133) with the gmx mdmat program. Principal-component analysis (PCA) captures the major fluctuations in the protein conformations during the MD simulation. PCA was performed using gmx covar and gmx anaeig programs, where the covariance matrix was first calculated for the C-α atom of the TLR-vaccine chain. After diagonalizing this covariance matrix, the eigenvectors and eigenvalues were scored for each snapshot collected at 10 ps. The eigenvectors show the motion path, while the eigenvalues show the mean square fluctuation. Two principal components (PC1 and PC2) were selected for the analysis.

These two PCs were further used to calculate and analyze Gibb’s free energy landscape (Gibb’s FEL), the stable lowest energy state (134), with the gmx sham program. In Gibb’s FEL plots, the deep valleys represent the lowest energy states, while the boundaries between the deep valleys show intermediate conformations. The secondary structure changes in TLR and vaccine structure were analyzed using the protein secondary structure (DSSP) program (135, 136). The motions of the TLR chain and vaccine chain in each system and the motions in segments of each chain were analyzed with dynamical cross-correlation matrix (DCCM) analysis (137).

**(ii) MM-PBSA calculations.** The trajectories extracted at every 2 ns from 75 ns to 100 ns of MD simulation were subjected to Poisson Boltzmann surface area continuum solvation (MM-PBSA) calculations (138–140) to derive the binding free energy estimates between TLR chain and vaccine chain.

The protein structures were rendered in ChimeraX (138), PyMOL (139), and VMD (140), and graphs were obtained from XMGRACE (141). Gibb’s FEL plots were generated using the Python-based Matplotlib package (142). The DCCM analysis was performed in the R statistical program (143) with the Bio3D package (144).

### Immune simulation.

In this study, the immune simulation of EBV was conducted using the C-ImmSim online server (http://150.146.2.1/C-IMMSIM/index.php), which generates a real immune interaction prediction just like in real life (145). The server applies machine learning methods and position-specific scoring matrix (PSSM) techniques to predict adaptive immunity generation and host epitope-specific immune interactions. The immune simulation kept all parameters default, except for time steps (set at 1, 84, and 170), and the number of simulation steps was set at 1,050. The recommended dosage for the vaccine stood to three injections at the interval of 4 weeks each, which is also the ideal interval between doses for every commercially available vaccine (146).

### Codon adaptation, *in silico* cloning within the E. coli system, and prediction of the vaccine mRNA structure.

For codon adaptation and *in silico* cloning, reverse translation of the query protein was conducted to find the potential DNA sequence that would transcribe our peptide vaccine (147). The DNA was adapted and incorporated into the desired organism so that the organism could effectively translate the desired protein by using the codons of the adapted DNA sequence. The codon adaptation of the designed vaccine protein was conducted using the Java Codon Adaptation Tool or JCat server (http://www.jcat.de/) (146). The eukaryotic E. coli strain K-12 was selected as the desired organism while avoiding the rho-independent transcription terminators, prokaryotic ribosome-binding sites, and EaeI and StyI cleavage sites of restriction enzymes. The Eae1 and StyI restriction sites were combined, taking the optimized DNA sequence at the N-terminal and C-terminal sites, respectively. Afterward, for inserting the newly adapted DNA sequence between the EaeI and StyI restriction sites of the pETite vector (Lucigen, USA), SnapGene restriction cloning software was used (88). Two tags, namely, the SUMO tag and 6×His tag present in the pETite vector within its DNA sequence, facilitated the solubilization and effective affinity purification of the recombinant protein (148).

Following the successful conduction of *in silico* cloning, two servers (i.e., Mfold [http://unafold.rna.albany.edu/?q=mfold] and RNAfold [http://rna.tbi.univie.ac.at/cgi-bin/RNAWebSuite/RNAfold.cgi]) were used to generate the mRNA secondary structure of the vaccine protein. These servers predict each generated mRNA secondary structure’s minimum free energy (G kcal/mol). Lower minimum free energy indicates a more stably folded mRNA structure (149–152). The optimized DNA sequence from the JCat server was first converted to RNA sequence by the DNA<->RNA->Protein tool (https://biomodel.uah.es/en/lab/cybertory/analysis/trans.htm). Afterward, the RNA sequence was collected from the tool and utilized in the Mfold and RNAfold servers for prediction, keeping all the parameters default.
